# Sarcopenia knowledge of geriatric rehabilitation patients is low while they are willing to start sarcopenia treatment: EMPOWER‐GR

**DOI:** 10.1002/jcsm.13372

**Published:** 2023-12-20

**Authors:** Laure M.G. Verstraeten, Amir Mashni, Janneke P. van Wijngaarden, Carel G.M. Meskers, Andrea B. Maier

**Affiliations:** ^1^ Department of Human Movement Sciences @AgeAmsterdam, Vrije Universiteit Amsterdam, Amsterdam Movement Sciences van der Boechorststraat 7 Amsterdam 1081 BT The Netherlands; ^2^ Danone Nutricia Research Utrecht The Netherlands; ^3^ Department of Rehabilitation Medicine Amsterdam University Medical Centre, Amsterdam Movement Sciences Amsterdam The Netherlands; ^4^ Department of Medicine and Aged Care @AgeMelbourne, The Royal Melbourne Hospital, The University of Melbourne Parkville Victoria Australia; ^5^ Healthy Longevity Translational Research Program, Yong Loo Lin School of Medicine National University of Singapore Singapore; ^6^ Centre for Healthy Longevity @AgeSingapore, National University Health System Singapore

**Keywords:** aged, knowledge, rehabilitation, sarcopenia, survey

## Abstract

**Background:**

Sarcopenia is prevalent in 20–50% of geriatric rehabilitation inpatients and is associated with functional dependence and mortality. The aim is to assess knowledge of geriatric rehabilitation inpatients on sarcopenia and their willingness and perceived barriers to start treatment.

**Methods:**

Enhancing Muscle POWER in Geriatric Rehabilitation (EMPOWER‐GR) is an observational cohort of geriatric rehabilitation inpatients in Amsterdam, the Netherlands. Knowledge of sarcopenia, willingness and perceived barriers to treatment were assessed with a survey among inpatients. Importance of and self‐perceived muscle health were rated using a visual analogue scale from 0 to 10. Descriptive statistics were used.

**Results:**

Inpatients' (*n* = 157, 59.9% female) mean age was 80.5 years (SD 7.3). Sarcopenia (European Working Group on Sarcopenia in Older People 2) prevalence was 21.7%. Five inpatients (3.2%) had heard of sarcopenia and had knowledge of its definition. Median muscle health was rated as 6 (interquartile range: 4–7). After explanation of treatment options, 67.1% were willing to start resistance exercise training (RET), 61.1% a high‐protein diet and 55.7% oral nutritional supplements (ONS). Inpatients with sarcopenia were less willing (51.6%) to start a high‐protein diet compared with inpatients without sarcopenia (77.8%) (*P* = 0.002); there was no difference for RET and ONS. Most reported barriers to treatment were ONS dislike (17.0%), too many other health issues (13.6%), doubts about treatment effectiveness/importance (12.9%) and RET intensity/difficulty (10.2%).

**Conclusions:**

Knowledge of sarcopenia was low, while the majority of inpatients showed willingness to start treatment. A dislike of ONS, RET difficulty and too many other health issues may reduce willingness to start treatment. Education is important to increase sarcopenia‐related health issues in geriatric rehabilitation inpatients.

## Introduction

Sarcopenia, age‐related low muscle strength and muscle mass,^1^ is prevalent in 37% of hospitalized older adults^2^ and 56% of geriatric rehabilitation inpatients.^3^ Physical inactivity and malnutrition are modifiable risk factors of sarcopenia,^1^ which is associated with functional dependence,^4^ falls and fractures,^5^ and mortality^6^ in older adults. Resistance exercise training (RET) and protein supplementation were shown to increase muscle mass and muscle strength.^7^ Clinical trials report good compliance to such intervention in community‐dwelling older adults and hospitalized patients,^8^ but education is key to improve patient compliance in clinical practice and therewith response to treatment.^9^, ^10^ Moreover, better knowledge on sarcopenia and the importance of muscle health among older adults and patients may help mitigate risk factors of sarcopenia and thereby hospitalization and geriatric rehabilitation admission.^11^

Although the large majority of healthcare professionals reports to know the term sarcopenia, diagnosis and treatment implementation in clinical practice is lacking due to a lack of access to equipment, knowledge, time and priority.^12^, ^13^ Contrastingly, sarcopenia is largely unknown among the general public as the term is poorly searched on Google compared with other age‐related diseases like dementia and osteoporosis.^14^ Also, a majority of community‐dwelling older adults lack knowledge on sarcopenia; common barriers to treatment include time constraints, costs and dislike to visiting a healthcare professional.^15^ Geriatric rehabilitation provides a window of opportunity for starting treatment of sarcopenia given its multidisciplinary approach with interventions to optimize functional recovery.^16^ However, sarcopenia knowledge, and treatment willingness and barriers are unknown in this population, which is important to guide and improve diagnosis and implementation of treatment in clinical practice.

The aim of this study was to assess knowledge of geriatric rehabilitation inpatients on sarcopenia, as well as their willingness and perceived barriers to start treatment.

## Methods

### Study design and population

Enhancing Muscle POWER in Geriatric Rehabilitation (EMPOWER‐GR) is an observational, longitudinal cohort of geriatric rehabilitation inpatients admitted to a geriatric rehabilitation ward of the care provider Cordaan (location Hof van Sloten, Amsterdam, the Netherlands). A Comprehensive Geriatric Assessment (CGA) was completed within 4 days of admission by physicians, nurses, physiotherapists, occupational therapists and researchers. Inpatients were excluded if younger than 65 years, unable to provide informed consent (assessed by the physician in charge of admission) or to communicate in Dutch, receiving palliative care at admission or in contact isolation. Methodology details are provided in the protocol article.^17^ The Medical Ethics Committee of the Amsterdam UMC (location VUmc) gave exemption for the study (2020.350). Written informed consent was provided from all inpatients prior to inclusion. The study was performed in accordance with the Dutch Medical Research Involving Human Subjects Act and the Declaration of Helsinki. Inpatients admitted from 16 November 2020 to 14 October 2021 were eligible for inclusion. Of the 297 inpatients admitted, 72 inpatients were excluded and 25 refused to consent; a total of 200 inpatients were included in the EMPOWER‐GR cohort.

### Inpatient characteristics

All assessments were performed within 4 days of admission to geriatric rehabilitation. Age, sex, primary reason for acute hospital admission and medication use were retrieved from inpatient files. Living situation, education, profession, use of walking aid and falls in the year prior to admission were collected with a survey. Morbidities were summarized by physicians using the 56‐point Cumulative Illness Rating Scale (CIRS).^18^ Cognitive impairment was defined as a dementia diagnosis reported in medical records or CIRS or a Standardized Mini‐Mental State Examination (SMMSE) score < 24 points or a Montreal Cognitive Assessment (MoCA) score < 26 points, performed by a physician or a researcher.^19^

Nurses performed anthropometric measurements. Standing height was measured without footwear up to the nearest 0.1 cm with a stadiometer. When unable to stand, knee height was measured using a sliding calliper between knee and ankle joints positioned at 90°; height was estimated with the Chumlea equation for Caucasians.^20^ Weight was measured without shoes and heavy clothing on a calibrated weighing chair or passive lift to the nearest 0.1 kg. Body mass index (BMI) was calculated by dividing body weight by height squared (kilograms per square metre). Malnutrition risk was assessed by a researcher with the Mini Nutritional Assessment Short Form (MNA‐SF) on a scale from 0 to 14.^21^ Malnutrition diagnosis, assessed by a researcher, was based upon the Global Leadership Initiative on Malnutrition (GLIM) criteria, with malnutrition diagnosis occurring when at least one phenotypic criterion (low BMI, reduced muscle mass or non‐volitional weight loss) and aetiologic criterion (any chronic gastrointestinal condition adversely impacting food assimilation or absorption, disease burden or reduced food intake) were separately met.

Functional performance at admission and pre‐acute hospital admission was assessed by an occupational therapist using the Katz index for activities of daily living (ADL) (0–6 points) and the Lawton and Brody scale for instrumental ADL (IADL) (0–8 points).^22^, ^23^ Physical performance was assessed by a physiotherapist with the Short Physical Performance Battery (SPPB), including the balance test, 4‐m walk test and chair stand test, with a score from 0 to 12 points.^24^ Handgrip strength (HGS) was measured with a handheld hydraulic dynamometer by a physiotherapist or a researcher (JAMAR, Sammons Preston, Inc., 119 Bolingbrook, IL, USA) in a sitting position, elbow bent at 90° to the body, exerting maximum force. HGS was measured six times, alternating for both hands, and the maximum value was used for analysis,^25^ expressed in kilograms.

Muscle mass was measured by a researcher using direct‐segmental multi‐frequency bio‐electrical impedance analysis (DSM‐BIA; InBody S10, Biospace Co., Ltd, Seoul, South Korea) in a supine position. DSM‐BIA was not performed in inpatients with (1) an electronic internal medical device or implant such as a pacemaker; (2) plasters or bandages that interfered with the placement of the electrodes; and (3) an amputation. Muscle mass was expressed as appendicular lean mass (ALM) in kilograms, and ALM index (ALMI; kilograms per square metre) was calculated by dividing ALM (kilograms) by height squared (square metres). Sarcopenia (low muscle strength and low muscle mass) diagnosis was based on the second European Working Group on Sarcopenia in Older People (EWGSOP2) definition.^1^ Low muscle strength was defined as HGS of <27 and <16 kg for males and females, respectively, and low muscle mass as ALMI of <7.0 and <5.5 kg/m^2^. Inpatients with normal muscle strength were classified as no sarcopenia, inpatients with low muscle strength but normal muscle mass as probable sarcopenia and inpatients with low muscle strength and low muscle mass as confirmed sarcopenia.

### Survey

The survey was undertaken by a researcher within the first week of admission in patients included in the EMPOWER‐GR cohort. Patients who were unable to answer the survey as judged by the researcher were excluded (*Figure* *S1*). The survey was designed based on a previously applied survey on sarcopenia knowledge in community‐dwelling older adults.^15^ The survey was tested by the authors, one researcher who was not involved in the study and five older adults to ensure face validity, readability and clarity of content. The survey was adapted based on the comments provided. The survey included questions on (1) knowledge of the term sarcopenia and ‘muscle poverty’ (Dutch: spierarmoede), after which the EWGSOP2 definition of sarcopenia was introduced^1^; (2) self‐perceived muscle health and knowledge of the importance of muscle health for overall health, physical activity, nutrition and sarcopenia seriousness (rating scale from 0 to 10, with 0 representing *not important at all*, *not serious at all* or *very poor* and 10 representing *very important*, *very serious* or *very good*); (3) knowledge of the causes, consequences and treatment for sarcopenia, after which the recommended treatment for sarcopenia with RET, high‐protein diet and oral nutritional supplements (ONS) was introduced^26^; (4) willingness to start sarcopenia treatment and perceived barriers (predefined answers/other) to sarcopenia treatment; and (5) willingness to prevent sarcopenia. The full survey is shown in *Appendix* *S2*.

### Statistical analysis

Patient characteristics and survey answers were presented using descriptive statistics. Median values and interquartile range (IQR) were reported for non‐normally distributed variables. Categorical variables were reported as a frequency and percentage. Incomplete surveys were included; surveys were excluded if answers to the first questions on sarcopenia knowledge were lacking. For the Question Numbers 22, 23 and 26 (*Appendix* *S2*), two individual researchers independently recategorized the answers reported under ‘other’ when appropriate. All open‐text answers provided under ‘other’ are shown in *Table* *S3*. Analyses were performed on a group level and stratified by sarcopenia status (no sarcopenia vs. probable/confirmed sarcopenia), musculoskeletal condition (reported in CIRS vs. no condition), cognitive impairment (impairment vs. no impairment), professional background (health related vs. other), education (higher education vs. lower) and living situation (alone vs. not alone). Non‐parametric Mann–Whitney *U* test was performed to assess differences between the two groups for non‐normally distributed continuous variables and *χ* ^2^ test of homogeneity for categorical variables. *P*‐values of <0.05 were considered statistically significant. Statistical analyses were performed using the Statistical Package for the Social Sciences (IBM SPSS Advanced Statistics 27.0, Armonk, NY, USA: IBM Corp).

## Results

Survey answers were available for 157 of the 200 inpatients of the EMPOWER‐GR cohort (*Figure* *S1*); their characteristics are shown in *Table* *1*. Mean age was 80.5 years (SD 7.3), and 59.9% were female. Prevalence of malnutrition and sarcopenia was 73.0% and 21.7%, respectively. Excluded inpatients due to incomplete surveys (*n* = 43) had a higher CIRS score and prevalence of cognitive impairment and lower functional performance compared with the survey respondents (*Table* *S4*).

**Table 1:** Inpatient characteristics at admission to geriatric rehabilitation

Characteristics	*n*	Total
Age (years), mean ± SD	157	80.5 ± 7.3
Female, *n* (%)	157	94 (59.9)
Living alone, *n* (%)	155	109 (70.3)
Education (years)	145	10 [9–14]
Reported falling in previous year, *n* (%)	154	105 (68.2)
Reason for acute admission, *n* (%)	157	
Musculoskeletal		62 (39.5)
Neurological		28 (17.8)
Infection		22 (14.0)
Cardiac		12 (7.6)
Cancer		11 (7.0)
Gastrointestinal		10 (6.4)
Other		12 (7.6)
CIRS score (0–56) (points)	157	9 [7–13]
Cognitive impairment, *n* (%)	157	46 (29.3)
SMMSE score (0–30) (points)	152	27 [23–28]
Body mass index (kg/m^2^)	156	25.7 [23.1–29.5]
Malnutrition (GLIM), *n* (%)	137	100 (73.0)
Katz‐ADL score (0–6) (points)^a^	157	3 [2–6]
Premorbid^b^		6 [6–6]
Lawton and Brody‐IADL score (0–8) (points)^a^	154	3 [2–4]
Premorbid^b^		7 [6–8]
Premorbid use of walking aid, *n* (%)	156	87 (55.8)
Unable to walk at admission, *n* (%)^c^	157	63 (43.1)
Premorbid^b^		2 (1.3)
SPPB score (0–12) (points)^d^	156	2 [0–5]
Handgrip strength (kg)	157	17.5 [13.0–24.0]
Female	94	15.0 [12.0–20.0]
Male	63	23.0 [17.0–29.0]
Appendicular lean mass index (kg/m^2^)	132	6.00 [6.73–7.82]
Female	80	5.77 [6.37–7.17]
Male	52	7.41 [6.55–8.38]
Sarcopenia (EWGSOP2), *n* (%)	138	
Probable		51 (37.0)
Confirmed		30 (21.7)

*Note*: All data are presented as medians [interquartile range], unless noted otherwise. Abbreviations: (I)ADL, (instrumental) activities of daily living; CIRS, Cumulative Illness Rating Scale; EWGSOP2, European Working Group on Sarcopenia in Older People revised definition; GLIM, Global Leadership Initiative on Malnutrition; SD, standard deviation; SMMSE, Standardized Mini‐Mental State Examination; SPPB, Short Physical Performance Battery.

^a^ An overview of each (I)ADL is provided in *Table* *S7*.

^b^ Two weeks prior to acute hospitalization.

^c^ Functional Ambulation Classification (FAC) ≤ 2 points.

^d^ Balance test: 0 [0–4] points; 4‐m walk test: 0.49 [0.30–0.65] m/s, *n* = 86 unable to perform the test; chair stand test: 20.4 [15.1–27.3] s, *n* = 123 unable to perform the test.

### Sarcopenia knowledge

**Figure 1:**
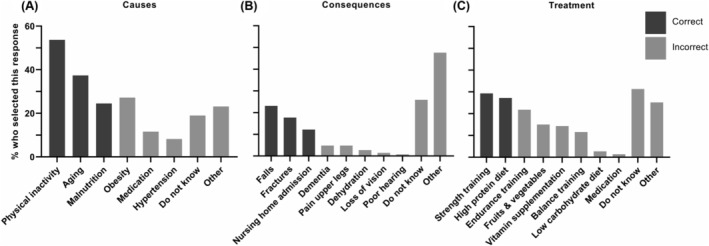
Knowledge about the causes (A), consequences (B) and treatment (C) of sarcopenia in geriatric rehabilitation inpatients, after explanation of what sarcopenia is (*n* = 147). Multiple answers were possible.

Five inpatients (3.2%) stated to have heard of the term ‘sarcopenia’ and 25.5% (*n* = 40) of the term ‘muscle poverty’ (Dutch: ‘spierarmoede’) (*Table* *2*). An overview of answers provided to define ‘muscle poverty’ is shown in *Table* *S5*. The majority of inpatients (*n* = 94, 63.5%) selected protein as important for muscle health; this percentage was higher in inpatients without sarcopenia (75.9%), without cognitive impairment (72.0%), with a health‐related professional background (90.9%) and with a higher education (77.3%) compared with inpatients with sarcopenia (56.4%) (*P* = 0.017), with cognitive impairment (41.5%) (*P* < 0.001), without a health‐related professional background (60.6%) (*P* = 0.046) and without a higher education (58.8%) (*P* = 0.033), respectively (*Table* *S6*). Inpatients stated that muscle mass starts to decline at 60 years (IQR: 50–70). Physical inactivity (*n* = 79, 53.7%) and aging (*n* = 55, 37.4%) were most selected as causes of sarcopenia (*Figure* *1* *A*); ‘do not know’ (*n* = 38, 25.9%), falls (*n* = 34, 23.1%) and fractures (*n* = 26, 17.7%) most selected as consequences of sarcopenia (*Figure* *1* *B*); and ‘do not know’ (*n* = 46, 31.3%), strength training (*n* = 43, 29.3%) and high‐protein diet (*n* = 40, 27.2%) most selected as treatment of sarcopenia (*Figure* *1* *C*).

**Table 2:** Sarcopenia knowledge in geriatric rehabilitation inpatients

	*n*	Total
Heard of term sarcopenia	157	5 (3.2)
Knows what sarcopenia is	157	5 (3.2)
Identifies sarcopenia as a disease of	150	
Muscle tissue		13 (8.7)
Brain tissue		3 (2.0)
Other (fat tissue, heart, bones and joints)		4 (2.7)
Do not know		130 (86.7)
Heard of term ‘muscle poverty’	157	40 (25.5)
Nutrients important for muscle health^a^	148	
Protein		94 (63.5)
Sugar		13 (8.8)
Fat		13 (8.8)
Energy		26 (17.6)
Vitamins		54 (36.5)
Minerals		28 (18.9)
Do not know		36 (24.3)
Age when muscle mass begins to decline (years)	120	60 [50–70]
Prevalence of sarcopenia in GR inpatients^b^	147	
<10%		3 (2.0)
10–20%		0 (0.0)
20–30%		1 (0.7)
30–40%		15 (10.2)
40–50%		13 (8.8)
>50%		40 (27.2)
Do not know		75 (51.0)

*Note*: All values are reported as *n* (%) or median [interquartile range]. Abbreviation: GR, geriatric rehabilitation.

^a^ Multiple answers were possible.

^b^ After explanation of what sarcopenia is.

**Figure 2:**
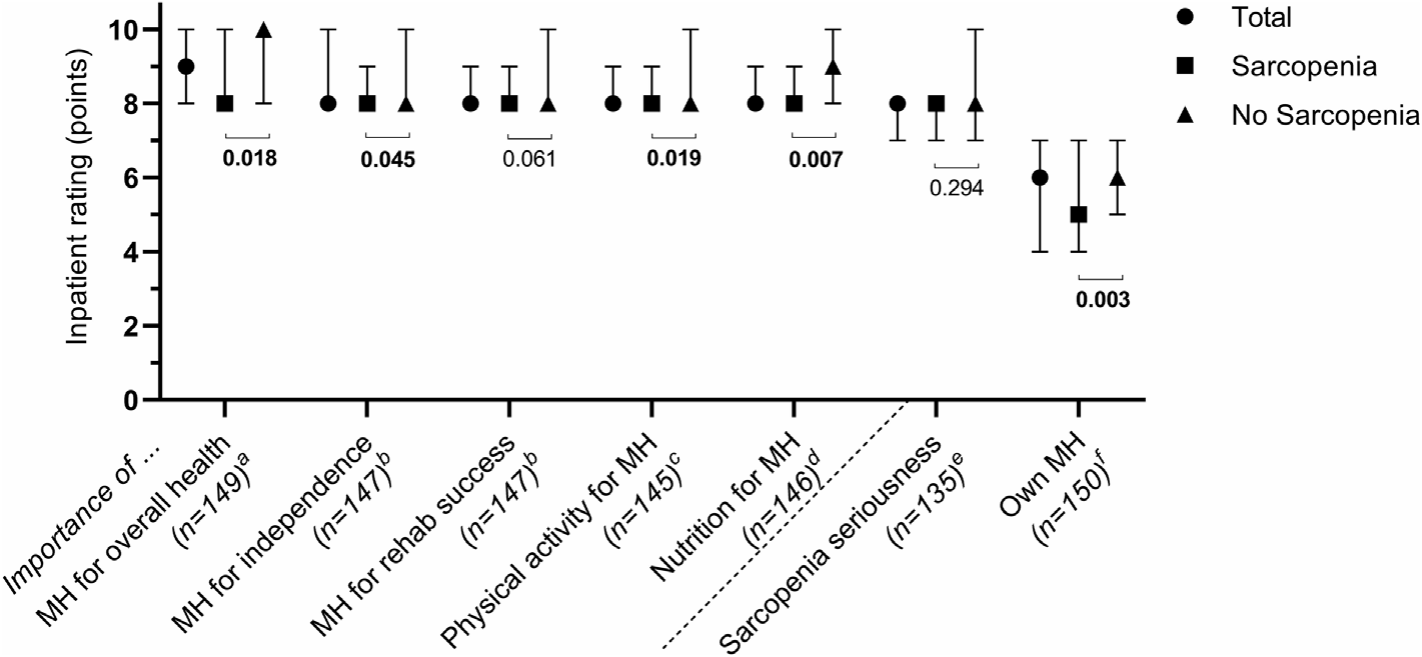
Importance ratings of muscle health (MH), perception of seriousness of sarcopenia and rating of own MH in geriatric rehabilitation inpatients, stratified by sarcopenia diagnosis. Rating scale was from 0 to 10, with 0 representing *not important at all*, *not serious at all* or *very poor* and 10 representing *very important*, *very serious* or *very good* for importance of, sarcopenia seriousness and rating of own MH, respectively. Data are presented as median and interquartile range. ^a^Sarcopenia (*n* = 93) and no sarcopenia (*n* = 56). ^b^Sarcopenia (*n* = 92) and no sarcopenia (*n* = 55). ^c^Sarcopenia (*n* = 92) and no sarcopenia (*n* = 53). ^d^Sarcopenia (*n* = 92) and no sarcopenia (*n* = 54). ^e^Sarcopenia (*n* = 84) and no sarcopenia (*n* = 51). ^f^Sarcopenia (*n* = 94) and no sarcopenia (*n* = 56).

The importance of muscle health for overall health, independence and rehabilitation success were rated by inpatients as 9 (IQR: 8–10), 8 (IQR: 8–10) and 8 (IQR: 8–9), respectively; seriousness of sarcopenia was rated as 8 (IQR: 7–8). The median rating of own muscle health was 6 (IQR: 4–7) (*Figure* *2*). Inpatients with sarcopenia had significantly lower ratings of own muscle health (*P* = 0.003); importance of muscle health for overall health (*P* = 0.018) and independence (*P* = 0.045); and importance of physical activity (*P* = 0.019) and nutrition (*P* = 0.007) for muscle health compared with inpatients without sarcopenia (*Figure* *2*).

### Willingness and barriers to sarcopenia treatment

The majority of inpatients (*n* = 125, 85.6%) were willing to start treatment for sarcopenia if diagnosed (*Table* *3*). After explanation of treatment options, 67.1% (*n* = 100), 61.1% (*n* = 91) and 55.7% (*n* = 83) were willing to start RET, a high‐protein diet and ONS, respectively; 10.1% (*n* = 15) selected none of the treatment options. Fewer patients with sarcopenia (51.6%) and more patients with a health‐related professional background (90.9%) were willing to start a high‐protein diet compared with patients without sarcopenia (77.8%) (*P* = 0.002) and without a health‐related professional background (90.9%) (*P* = 0.030), respectively; more patients with a musculoskeletal condition (71.3%) were willing to start RET compared with patient without a condition (52.9%) (*P* = 0.045; *Table* *S6*). When inpatients were specifically asked if they would be able and willing to start RET now, 58.4% (*n* = 87) responded affirmatively while 31.3% (*n* = 20) who answered ‘no’ (*n* = 62) stated it was too intensive. The most reported barriers to treatment were a dislike of ONS (*n* = 25, 17.0%) (in general, no specific type of ONS mentioned), too many other health issues (*n* = 20, 13.6%), doubts of treatment effectiveness and/or importance (*n* = 19, 12.9%), dislike and/or difficulty to adjust diet (*n* = 16, 10.9%) and RET being too intense/difficult (*n* = 15, 10.2%). No barriers were reported by 34 inpatients (23.1%). The majority of inpatients were willing to prevent sarcopenia by increasing physical activity (*n* = 83, 57.2%), activities that increase muscle strength (*n* = 76, 52.4%) and protein intake (*n* = 77, 53.1%). More patients with a musculoskeletal condition (57.1%) were willing to increase activities that increase muscle strength compared with patients without a condition (36.4%) (*P* = 0.036), and more patients without a higher education (35.4%) were not willing to prevent sarcopenia compared with patients with a higher education (15.9%) (*P* = 0.018; *Table* *S6*).

**Table 3:** Willingness to start sarcopenia treatment and barriers in geriatric rehabilitation inpatients, after explanation of what sarcopenia is

	*n*	Total
Willing to start treatment if sarcopenia diagnosed		
Before explaining what treatment is	146	125 (85.6)
After explaining what treatment is^a^	149	
RET 3×/week for 3 months		100 (67.1)
ONS 2×/day for 3 months		83 (55.7)
High‐protein diet on a daily basis		91 (61.1)
None		15 (10.1)
Willing and able to take part in RET now	149	87 (58.4)
If no, why?^a^	62	
Too intensive		20 (31.3)
Too difficult		10 (15.6)
Could be harmful		2 (3.1)
Other		39 (60.9)
Not motivated		8 (12.9)
Not important/not necessary		11 (17.7)
Health issues/too tired		13 (21.0)
Barriers to sarcopenia treatment^a^	147	
Takes too much time		6 (4.1)
Too many other health issues		20 (13.6)
Dislike ONS		25 (17.0)
Dislike physical activity		9 (6.1)
No supervision for RET when going home		9 (6.1)
Healthcare provider too far away after discharge		1 (0.7)
Treatment expensive		8 (5.4)
Consequences not severe enough		2 (1.4)
Dislike to go to healthcare provider		2 (1.4)
Other		77 (52.4)
Dislike/difficulty to adjust diet		16 (10.9)
Dislike RET specifically		4 (2.7)
RET too intense/difficult		15 (10.2)
Dislike/difficulty to leave home		5 (3.4)
Doubts about treatment effectiveness/importance		19 (12.9)
No barriers		34 (23.1)
Taking ONS now or in the past	147	62 (42.2)
Opinion on ONS^a^	145	
Useful to increase nutritional intake		14 (9.7)
Good protein source		24 (16.6)
High in calories		5 (3.4)
Contributes to treatment		41 (28.3)
Extra medication		5 (3.4)
Does not contribute to treatment		9 (6.2)
Do not know what ONS is		27 (18.6)
Other		65 (44.8)
Does not taste good/too sweet		21 (14.5)
No opinion/do not know what they are used for		21 (14.5)
Willing to prevent sarcopenia^a^	145	
Yes, increase physical activity		83 (57.2)
Yes, increase activities increasing muscle strength		76 (52.4)
Yes, increase protein intake		77 (53.1)
No		43 (29.7)
Patient suspects to have sarcopenia	141	51 (36.2)

*Note*: All values are reported as *n* (%). Abbreviations: ONS, oral nutritional supplements; RET, resistance exercise training.

^a^ Multiple answers were possible.

## Discussion

In a cohort of geriatric rehabilitation inpatients, knowledge of sarcopenia was very low (3%) and knowledge of the causes, consequences and treatment of sarcopenia was limited. Perceived importance of muscle health for overall health, independence and rehabilitation success was high. More than half of inpatients were willing to start RET, ONS and/or a high‐protein diet to treat sarcopenia. Most stated barriers to treatment included a dislike of ONS, RET difficulty, too many other health issues and doubts about treatment effectiveness and/or importance.

### Sarcopenia knowledge

The knowledge of sarcopenia was lower in geriatric rehabilitation inpatients compared with community‐dwelling older adults in the Netherlands, of whom 17% had heard of sarcopenia and 9% reported to know the definition of sarcopenia.^15^ The percentage of participants who had heard of the Dutch term for ‘muscle poverty’ (26%) was higher and comparable in both cohorts.^15^ This is in accordance with previous literature stating that native language informs and contributes to health literacy within a society.^27^ Nevertheless, awareness of the term sarcopenia besides muscle poverty is important for adequate implementation in clinical practice and adherence to treatment. Also, knowledge of other musculoskeletal conditions with similar prevalence such as osteoporosis is higher. A study using Google Trends showed that sarcopenia is poorly searched compared with osteoporosis^14^ and that knowledge of osteoporosis is estimated to be over 80% among young women, which is much higher compared with the present study.^28^ Similarly, the majority of healthcare professionals reports lacking knowledge to diagnose and treat sarcopenia,^12^ while 83% of physicians felt competent in managing osteoporosis.^29^

The age at which inpatients thought muscle mass to start to decline was higher compared with community‐dwelling older adults (46 years old), while the decline may begin as early as age 30 years.^30^ Also, more than one quarter of inpatients were unsure of the consequences and treatment for sarcopenia, although a larger proportion identified physical inactivity and aging as causes of sarcopenia compared with community‐dwelling older adults.^15^ In a survey assessing knowledge about protein in community‐dwelling older adults (*n* = 1825), 89.3% agreed that protein is needed in the diet to repair bones and muscles,^31^ which is higher compared with the present survey. Although knowledge of the importance of protein was relatively high in patients with a health‐related professional background and higher education, only half of inpatients with sarcopenia identified protein as important for muscle health. Better awareness of muscle mass decline at young age and awareness of the importance of RET and protein for muscle health are key to enable sarcopenia prevention and treatment.^32^ Healthcare professionals such as general practitioners, physiotherapists and dietitians may play a key role in spreading this message.

Inpatients recognized the importance of muscle health, and physical activity and nutrition for muscle health, although the ratings were lower in patients with sarcopenia and also lower (approximately 1 point out of 10) compared with community‐dwelling older adults.^15^ In the latter study, participants attended health education events and may therefore have been more health conscious.^15^ Similarly, inpatients with sarcopenia are likely to be less healthy compared with inpatients without sarcopenia.^33^ Lower self‐perceived muscle health compared with community‐dwelling older adults,^15^ especially in inpatients with sarcopenia, shows that inpatients may be aware of their poorer muscle health.

### Willingness and barriers to sarcopenia treatment

Overall, willingness to start treatment was lower compared with community‐dwelling older adults.^15^ This may be explained by a higher number of reported barriers in the present cohort, including too many other health issues. Geriatric rehabilitation inpatients often suffer from multimorbidity, and sarcopenia is highly prevalent in patients with cardiovascular disease, diabetes and respiratory disease.^34^ Also, willingness to start RET was higher compared with ONS and high‐protein diet, which may be explained by a dislike of ONS and diet change. While patients with a health‐related professional background were more willing to start a high‐protein diet, patients with sarcopenia were less willing compared with patients with sarcopenia, which shows that education efforts may need to be tailored. Interestingly, readiness for RET dropped when inpatients were asked directly if they would be willing to start treatment immediately. This might indicate self‐serving bias, namely, that inpatients may not be willing to begin training when the time comes. This lack of willingness may also be attributed to inpatient perceived goals of the treatment as some inpatients did not think RET was necessary and more so wanted to focus on regaining mobility, which is in accordance with other studies in rehabilitation.^35^ More than 10% of inpatients reported doubts about treatment effectiveness/importance, a finding that has been suggested for other conditions as well and could also be implicated for a lack of motivation. Similarly, a survey in older adults showed that 45% of respondents did not think RET increases muscle mass and that 37% of respondents perceived walking to be more effective than RET to increase muscle strength.^36^ Multiple studies show that RET and muscle strength are important for gait speed and stability.^37^ To increase willingness for treatment, comprehensive patient education should be explored, as, for example, formal education programmes have led to improved outcomes for patients managing diabetes.^38^ The information provided to patients should include evidence on efficacy and benefits related to treatment as previous research shows that these are important enablers for RET and ONS consumption.^10^, ^11^ Future studies should evaluate the efficacy of education sessions to increase sarcopenia knowledge and treatment willingness, also on the long term as knowledge retention may be challenging. Also, support from healthcare professionals and encouragement from family could serve as enablers of sarcopenia treatment.^39^ Lastly, attitudes towards treatment could be improved with interactive and motivating RET sessions and varying forms of protein supplementation.

### Strengths and limitations

This is the first study examining inpatient knowledge of sarcopenia in geriatric rehabilitation. All measurements were conducted by a multidisciplinary team as part of a CGA with validated and standardized assessments appropriate to older patients. A limitation of this study is the use of a custom survey in absence of a validated sarcopenia knowledge survey. The survey, while extensive, may not have been specific enough to capture the entire range of barriers or reasons for not starting treatment as many inpatients still reported ‘other’ as a reason. To rectify this, researchers reclassified some of these survey responses.

## Conclusions

Geriatric rehabilitation inpatients have little knowledge about sarcopenia. In contrast, they saw muscle health as being very important and were aware of their poor muscle health. Although the majority of inpatients is willing to start some form of treatment, a dislike of ONS, RET difficulty, too many other health issues and doubts about treatment effectiveness/importance are likely to hinder their compliance to treatment. Future interventions should focus on increasing knowledge of sarcopenia and importance of adequate treatment for health. Further research is needed to study enablers of sarcopenia treatment among inpatients.

## Conflict of interest statement

Andrea B. Maier reports grants from Danone Nutricia Research during the conduct of the study. Janneke P. van Wijngaarden reports that she is an employee of Danone Nutricia Research. Laure M. G. Verstraeten, Amir Mashni and Carel G. M. Meskers declare that they have no conflicts of interest.

## Supporting information


**Figure S1.** Flowchart of data availability for the sarcopenia survey as part of the EMPOWER‐GR cohort.

**Appendix S2.** Survey.

**Table S3.** Open‐text answers to “other” options (translated from Dutch to English).

**Table S4.** Inpatient characteristics that did not complete the sarcopenia survey compared to the total EMPOWER‐GR cohort characteristics.

**Table S5.** Inpatients' perception of “muscle poverty” (Dutch translation: “spierarmoede”) (*n* = 40).

**Table S6.** A. Survey answers stratified by sarcopenia status, musculoskeletal condition and cognitive impairment. B. Survey answers stratified by professional background, education and living situation.

**Table S7.** Overview of Katz and Lawton and Brody scales at admission and pre‐hospital admission.
