# Supplementary material for: Sarcopenia knowledge of geriatric rehabilitation patients is low while they are willing to start sarcopenia treatment: EMPOWER‐GR
Source: J Cachexia Sarcopenia Muscle. 2023 Dec 20;15(1):352–60. doi: 10.1002/jcsm.13372 (PMC10834324; doi:10.1002/jcsm.13372)
Supplement: Supplementary file 1 — Figure S1. Flowchart of data availability for the sarcopenia survey as part of the EMPOWER‐GR cohort. [file JCSM-15-352-s002.docx]

**Supplementary Material 1.** Flowchart of data availability for the sarcopenia survey as part of the EMPOWER-GR cohort.
