# Supplementary material for: Sarcopenia knowledge of geriatric rehabilitation patients is low while they are willing to start sarcopenia treatment: EMPOWER‐GR
Source: J Cachexia Sarcopenia Muscle. 2023 Dec 20;15(1):352–60. doi: 10.1002/jcsm.13372 (PMC10834324; doi:10.1002/jcsm.13372)
Supplement: Supplementary file 2 — Appendix S2. Survey. [file JCSM-15-352-s001.docx]

**Survey**

| **Sarcopenia Awareness** | |
| --- | --- |
| **1. Have you ever heard about the term sarcopenia?** | |
| Yes | No |
| **2. Do you know what sarcopenia is?** | |
| Yes | No |
| **3. Can you please try to describe or suggest in your own words what sarcopenia is / could be:**  __________________________________________________________________________________________________________________________________________________________________________ | |
| **4. Sarcopenia is a disease of which organ/tissue?** | |
| Brain  Fat  Heart  Muscles | |
| Bones  Liver  Joints  Don’t know | |
| Eyes  Kidney  Lungs | |

| **Knowledge about “muscle poverty”** | |
| --- | --- |
| **5. Have you ever heard about the term “muscle poverty”?** | |
| Yes | No |
| **6. Can you please try to describe or suggest in your own words what “muscle poverty” is / could be:**  __________________________________________________________________________________________________________________________________________________________________________ | |

**We would now like to explain what sarcopenia is.**

Sarcopenia, also known as “muscle poverty”, is a combination of:

- low skeletal strength;
- low muscle quantity or quality;
- low physical performance.

Now, based on this information, please answer the following questions:

| **7. How important is muscle health for overall health?** | | | | | | | | | | | | | | |
| --- | --- | --- | --- | --- | --- | --- | --- | --- | --- | --- | --- | --- | --- | --- |
| **0** | **1** | **2** | **3** | | **4** | **5** | | **6** | **7** | | | **8** | **9** | **10** |
|  |  |  |  | |  |  | |  |  | | |  |  |  |
| 0 = Not important at all 10 = Very important | | | | | | | | | | | | | | |
| **8. At this moment, how would you rate the health of your muscles?** | | | | | | | | | | | | | | |
| **0** | **1** | **2** | **3** | | **4** | **5** | | **6** | **7** | | | **8** | **9** | **10** |
|  |  |  |  | |  |  | |  |  | | |  |  |  |
| 0 = Very poor health 10 = Very good health | | | | | | | | | | | | | | |
| **9. At older age, how important is muscle health for independence?** | | | | | | | | | | | | | | |
| **0** | **1** | **2** | **3** | | **4** | **5** | | **6** | **7** | | | **8** | **9** | **10** |
|  |  |  |  | |  |  | |  |  | | |  |  |  |
| 0 = Not important at all 10 = Very important | | | | | | | | | | | | | | |
| **10. How Important is muscle health for the rehabilitation outcome/success?** | | | | | | | | | | | | | | |
| **0** | **1** | **2** | **3** | | **4** | **5** | | **6** | **7** | | | **8** | **9** | **10** |
|  |  |  |  | |  |  | |  |  | | |  |  |  |
| 0 = Not important at all 10 = Very important | | | | | | | | | | | | | | |
| **11. How important is physical activity in maintaining or improving muscle health?** | | | | | | | | | | | | | | |
| **0** | **1** | **2** | **3** | | **4** | **5** | | **6** | **7** | | | **8** | **9** | **10** |
|  |  |  |  | |  |  | |  |  | | |  |  |  |
| 0 = Not important at all 10 = Very important | | | | | | | | | | | | | | |
| **12. How important is nutrition in maintaining or improving muscle health?** | | | | | | | | | | | | | | |
| **0** | **1** | **2** | **3** | | **4** | **5** | | **6** | **7** | | | **8** | **9** | **10** |
|  |  |  |  | |  |  | |  |  | | |  |  |  |
| 0 = Not important at all 10 = Very important | | | | | | | | | | | | | | |
| **13. Which nutrient(s) is/ are important for muscle health? (multiple answers possible)** | | | | | | | | | | | | | | |
| Sugar | | | | Protein | | | | | | | Fat | | | |
| Vitamins | | | | Total energy | | | | | | | Minerals | | | |
| Don’t know | | | |  | | | | | | |  | | | |
| **14. At what age do you think muscle mass generally starts to decline?** | | | | | | | | | | | | | | |
| years old | | | | | | | | | | | | | | |
| **15. What are the cause(s) for sarcopenia? (multiple answers possible)** | | | | | | | | | | | | | | |
| Aging | | | | | | | Obesity | | | | | | | |
| Headaches | | | | | | | Physical inactivity | | | | | | | |
| High blood pressure | | | | | | | Don’t know | | | | | | | |
| Malnutrition | | | | | | | Other,______________________________ | | | | | | | |
| Medication | | | | | | |  | | | | | | | |
| **16. What are the consequence(s) of having sarcopenia? (multiple answers possible)** | | | | | | | | | | | | | | |
| Admission to a nursing home | | | | | | | Loss of vision | | | | | | | |
| Dementia | | | | | | | Pain in the upper legs | | | | | | | |
| Dehydration | | | | | | | Poor hearing | | | | | | | |
| Falls | | | | | | | Don’t know | | | | | | | |
| Fractures | | | | | | | Other,______________________________ | | | | | | | |
| **17. How should sarcopenia be treated? (multiple answers possible)** | | | | | | | | | | | | | | |
| Balance training | | | | | | | Medication | | | | | | | |
| Eat more fruits and vegetables | | | | | | | Strength training | | | | | | | |
| Endurance training | | | | | | | Vitamin supplements | | | | | | | |
| High protein diet (meat, fish, nuts, dairy, eggs) | | | | | | | Don’t know | | | | | | | |
| Low carbohydrate diet (low bread, | | | | | | | Other,______________________________ | | | | | | | |
| pasta and rice consumption) | | | | | | |  | | | | | | | |
| **18. How serious do you think sarcopenia is?** | | | | | | | | | | | | | | |
| **0** | **1** | **2** | **3** | | **4** | **5** | | **6** | **7** | | | **8** | **9** | **10** |
|  |  |  |  | |  |  | |  |  | | |  |  |  |
| 0 = Not serious at all 10 = Very serious | | | | | | | | | | | | | | |
| **19. In patients such as yourself admitted to geriatric rehabilitation, How often do you think sarcopenia occurs?** | | | | | | | | | | | | | | |
| < 10 % | | | | 30-40 % | | | | | | Don’t know | | | | |
| 10-20 % | | | | 40-50 % | | | | | |  | | | | |
| 20-30 % | | | | > 50 % | | | | | |  | | | | |
| **20. If you would have sarcopenia, would you be willing to start treatment?** | | | | | | | | | | | | | | |
| Yes | | | | | | | No | | | | | | | |

**The prevalence of sarcopenia in geriatric rehabilitation patients is estimated at 40%. Sarcopenia or “muscle poverty” should be treated with a combination of:**

1. Muscle strength training at least 3 times a week for 3 months. This includes exercises with weights (e.g. dumbbells, elastic bands) to increase the strength of the muscles.
2. High protein diet daily for breakfast, lunch and dinner (meat, fish, dairy, nuts). Eventually also oral nutritional supplements to increase protein and vitamins intake (2 servings per day for 3 months).

**Consequences**: sarcopenia increases the risk of falls and hospitalization and decreases quality of life and the ability to perform activities such as bathing, dressing and eating independently.

| **21. What treatment would you be willing to start if you would have sarcopenia? (select all options you would be willing to start)** | | | | | | | | | | |
| --- | --- | --- | --- | --- | --- | --- | --- | --- | --- | --- |
| Muscle strength training 3 times a week for 3 months. | | | | | | | | | | |
| High protein diet daily for breakfast, lunch and dinner. | | | | | | | | | | |
| Oral nutritional supplements intake (2 servings per day) for 3 months. | | | | | | | | | | |
| None | | | | | | | | | | |
| **22. Which barriers could keep you from starting treatment for sarcopenia, if needed? (multiple answers possible)** | | | | | | | | | | |
| Treatment would take too much time | | | | | | | | | | |
| I do not have supervision to perform the muscle strength exercises when I go home | | | | | | | | | | |
| I dislike oral nutritional supplements | | | | | | | | | | |
| I do not like physical activity | | | | | | | | | | |
| I have too many other health issues that also require treatment | | | | | | | | | | |
| Healthcare professionals (physiotherapist/dietician) would be too far away from where I live when I go home | | | | | | | | | | |
| Treatment might be expensive | | | | | | | | | | |
| The consequences of sarcopenia are not severe enough to treat | | | | | | | | | | |
| I don’t like to go to a healthcare professional | | | | | | | | | | |
| Other, please specify ____________________ | | | | | | | | | | |
| I would not have any barriers for sarcopenia treatment | | | | | | | | | | |
| **23. Would you be able and willing to take part in high intensity muscle strength training?** | | | | | | | | | | |
| Yes | | | | | | | | | | |
| No, please specify why: | | | It is too intensive for me | | | | | | | |
| (multiple answers possible) | | | It is too difficult for me | | | | | | | |
|  | | | It could be harmful for me | | | | | | | |
|  | | | Other, please specify_________________________________ | | | | | | | |
| **24. Would you prefer to perform the high intensity strength training alone with the physiotherapist or in group with other rehabilitation patients?** | | | | | | | | | | |
| Alone | | | | In group | | | | | I do not mind | |
| **25. Have you already been taking oral nutritional supplements?** | | | | | | | | | | |
| No, I have never taken oral nutritional supplements | | | | | | | | | | |
| Yes, please specify: | | | I am currently taking oral nutritional supplements | | | | | | | |
|  | | | I was taking oral nutritional supplements at the hospital before admission to geriatric rehabilitation but I stopped | | | | | | | |
|  | | | I was taking oral nutritional supplements in the past | | | | | | | |
|  | | | Other, please specify_________________________________ | | | | | | | |
| **26. What is your general opinion of oral nutritional supplements? (multiple answers possible)** | | | | | | | | | | |
| Useful to increase nutritional intake | | | | | | Extra drug/medication | | | | |
| Good source of protein | | | | | | It does not contribute to my treatment | | | | |
| It contributes to my treatment | | | | | | I do not know what it is | | | | |
| High in calories | | | | | | Other, please specify_____________________ | | | | |
| **27. To which extent do you agree with these statements?**   \|  \| Completely agree \| Rather agree \| Rather disagree \| Completely disagree \| Do not know \| \| --- \| --- \| --- \| --- \| --- \| --- \| \| Oral nutritional supplements might help to increase nutritional intake \| 4 \| 3 \| 2 \| 1 \| 0 \| \| Oral nutritional supplements might be good for muscle health \| 4 \| 3 \| 2 \| 1 \| 0 \| \| Oral nutritional supplements might help improve physical activity \| 4 \| 3 \| 2 \| 1 \| 0 \| \| Oral nutritional supplements might help increase quality of my life \| 4 \| 3 \| 2 \| 1 \| 0 \| \| Oral nutritional supplements might help prevent or treat sarcopenia \| 4 \| 3 \| 2 \| 1 \| 0 \| | | | | | | | | | | |
| **28. If you do not have sarcopenia, would you be willing to prevent sarcopenia by improving your lifestyle? (multiple answers possible)** | | | | | | | | | | |
| Yes, I would increase physical activity | | | | | | | | | | |
| Yes, I would increase activities increasing muscle strength | | | | | | | | | | |
| Yes, I would increase protein intake | | | | | |  | | | | |
| No | | | | | |  | | | | |
| **29. Do you think you have sarcopenia?** | | | | | | | | | | |
| Yes | No | | | | | |  | | |  |
| **30. Have you previously been diagnosed with sarcopenia?** | | | | | | | | | | |
| Yes | | No | | |  | | |  | | |
| If yes, when have you been diagnosed (year) | | | | | | | | | | |
