# Supplementary material for: Sarcopenia knowledge of geriatric rehabilitation patients is low while they are willing to start sarcopenia treatment: EMPOWER‐GR
Source: J Cachexia Sarcopenia Muscle. 2023 Dec 20;15(1):352–60. doi: 10.1002/jcsm.13372 (PMC10834324; doi:10.1002/jcsm.13372)
Supplement: Supplementary file 3 — Table S3. Open‐text answers to “other” options (translated from Dutch to English). [file JCSM-15-352-s007.docx]

**Table S3. Open-text answers to “other” options (translated from Dutch to English)**

|  |
| --- |
| **Q15 What are the cause(s) for sarcopenia?** |
| Alcohol, unhealthy lifestyle (2x) |
| Bad food (2x) |
| Combination, multiple diseases |
| Damage due to use (2x) |
| Damage due to use, overload |
| Difficult to explain |
| Disease (9x) |
| Disease (MS-Parkinson) |
| Disease, covid |
| Little use of the muscles |
| Lying in bed due to illness |
| Moving less |
| No calcium |
| No intensive work |
| Not using muscles (2x) |
| Overall health |
| Poor nutrition (3x) |
| The thinking |
| Too much alcohol, poor nutrition |
| Weight loss (2x) |
|  |
| **Q16 What are the consequence(s) of having sarcopenia?** |
| Accelerated aging |
| Back pain |
| Becoming disabled |
| Becoming stiffer |
| Becoming too fat |
| Being sick, getting older |
| Able to do less |
| Climbing stairs less easily |
| Decline |
| Dependence |
| Dependence on others |
| Deterioration (2x) |
| Difficulty moving |
| Difficulty with daily activities, inability to move |
| Do less |
| Help needed for everything |
| Illness, not being able to do anything anymore |
| Immobility |
| Impediment to movement |
| Inability to do anything |
| Inability to walk (7x) |
| Inability to walk, wheelchair |
| Inability to walk/ stand |
| Less independent |
| Less mobile (4x) |
| Less mobile, collapsing |
| Less strength |
| Mobility |
| Move less |
| Move less easily |
| Movement poverty |
| Multiple Sclerosis |
| No longer moving forward |
| Not being able (2x) |
| Not being able to do anything anymore |
| Not being able to do everything anymore (2x) |
| Not being able to do everything yourself anymore |
| Not being able to do much |
| Not being active anymore |
| Not feeling well |
| Organs failure |
| Organs not working well |
| Paralysis |
| Passing away |
| Physical decline |
| Poor health, acceleration of decline |
| Poor health, depression |
| Poor mobility (2x) |
| Reduced mobility |
| Reduction of all bodily functions |
| Walk less |
| Walking less well |
| Walking poorly |
| Weak muscles |
| Wheelchair |
| Wheelchair bound |
| You cannot do anything anymore |
|  |
| **Q17 How should sarcopenia be treated?** |
| Accept |
| Cannot be treated |
| Diet and training |
| Eating healthier |
| Eating well |
| Exercise with physiotherapist |
| Exercises |
| General movement |
| General training |
| Good care |
| Gymnastic (2x) |
| Healthy food |
| Healthy lifestyle and stopping to smoke |
| Just move |
| Less fat |
| Move (3x) |
| Movement |
| Movement in general |
| Moving a lot (2x) |
| No idea |
| Not doable at older age, needs to start when young and healthy |
| Nothing, it is normal, impossible to build muscle mass |
| Nutrition |
| Physiotherapy (2x) |
| Physiotherapy |
| Swimming, gymnastic |
| Training in general |
| Training in general and diet |
| Walking (2x) |
| Walking training |
| Walking, healthy food |
| Yoga |
|  |
| **Q22 Which barriers could keep you from starting treatment for sarcopenia, if needed?** |
| After 3 months you will not be a new person |
| Already exercising daily |
| Already need to go a lot to the hospital, no time for 3x resistance training |
| At older age you need to do what you like, extra treatment is pointless |
| Bad mobility |
| Before sarcopenia I try to avoid overweight |
| Can not have too much protein because of kidney problems |
| Can not go independently to the fitness school and I have intestinal problems |
| Condition training nicer than resistance |
| COPD makes resistance training difficult en I do not feel like it anymore |
| Difficult to change diet |
| Difficult to change diet, I eat what I like |
| Difficult to go to the physiotherapist, someone would need to bring me |
| Do not feel like doing extra exercises, no diet because I already eat healthy |
| Do not feel like training, have done it a lot in the past but not needed anymore |
| Do not know, first try |
| Do not like diet |
| Do not like diet and body is tired |
| Do not like diet or training, too difficult |
| Do not like fitness schools |
| Do not like fitness schools, training must be nice |
| Do not like resistance training |
| Do not like to cook/ diet |
| Do not think I would benefit from extra nutrition, I eat cottage cheese, I am followed by a dietitian and have swallowing problems |
| Do not think it will help, must remain a pleasure |
| Do not think it would work, focus on walking |
| Do not want to travel outside |
| Doubts if I could do it |
| Find discipline/ motivation |
| Following a low salt diet and do not like dairy products |
| Heart problems |
| High protein diet will be expensive |
| I am already always active in the garden |
| I am too dependent now |
| I do not know (2x) |
| I do not want to follow a diet |
| I have become lazier |
| If I need to travel too far |
| Just do not feel like it, too old |
| Just want to be able to go home, but the rest is not possible anymore |
| Let nature takes its course |
| Lots to do myself |
| Lot of pain, needs prove it works |
| Lots of sugar in ONS |
| Maybe do not feel like it |
| Missing a long |
| No complicated diet, because otherwise lifestyle needs to be adjusted |
| No idea |
| No longer necessary at this age |
| Not mobile enough |
| Not needed |
| Not sure I see the point |
| Only being able to walk again is important |
| Only resistance training if proven it helps |
| ONS only if really needed, preference for training |
| Pacemaker (2x) |
| Pain, exhausted quickly |
| Prefer condition than resistance training, do not see the point of resistance training, more for young people |
| Resistance training may be too intensive |
| Resistance training difficult because of heart, do not like meat/fish |
| Resistance training difficult because of sternum/heart operation |
| Resistance training is not fun, dancing nicer, important it remains fun |
| Resistance training is too difficult |
| Resistance training is too much for me and do not feel like it |
| Resistance training too difficult after surgery, but would be motivated after with a personal trainer |
| Resistance training too difficult, but would try it, eat not meat/fish |
| Resistance training would be difficult because of bad shoulders |
| Short of breath, no energy, do not tolerate dairy products well |
| Still too weak at the moment |
| Subscription costs for fitness school |
| That it comes at the expense of other things |
| Tingling in hands/feet due to neck problems and bad knees |
| Too busy with rehabilitation, too much on my mind, now focusing on being able to walk to go home |
| Too difficult |
| Too much pain |
| Too old |
| Too old for 3x resistance training per week |
| Too old for resistance training |
| Too tired |
| Way too difficult for me |
| Weather conditions |
| Wondering if worth it at my age |
| Would do it but would find it annoying, not too much ONS because of renal function |
| Would need to be planned well, difficult to get out of home |
| Would prefer diet first and if very serious than ONS |
| Would prefer diet first and only if not sufficient than ONS |
|  |
| **Q23 Would you be able and willing to take part in high intensity muscle strength training. If no, why?** |
| At older age it is better to do what you enjoy, extra treatment does not make sense |
| Better walking exercises |
| Cannot move at the moment |
| Condition training better than resistance training |
| Difficult because of rheumatism |
| Do I really need to become 150 |
| Does not like resistance training, prefers other types of movement |
| Does not see the point |
| Doubts about effectiveness |
| Had a heart surgery, contraindication for resistance training |
| Has rheumatism |
| Heart condition |
| Heart problems |
| Learning to walk is the most important |
| Maybe later but too much now |
| No need for resistance training, only being able to walk well (2x) |
| No use |
| Not feeling like it (3x) |
| Not feeling like it, doing physiotherapy but only exercises I like |
| Not fun |
| Not needed |
| Not needed, does not have sarcopenia |
| Not needed, walking is most important |
| Not now, first rehabilitation |
| Pacemaker (2x) |
| Prefers working in the garden |
| Short of breath because of COPD |
| Too lazy |
| Too much effort |
| Too much shortness of breath |
| Too old (2x) |
| Too tired |
| Too tired now, maybe later |
| Want to determine how much I train |
|  |
| **Q26 What is your general opinion of oral nutritional supplements?** |
| A lot, do not know whether it contributes to treatment, take it because I must |
| Bad taste (6x) |
| Bad taste, but healthy |
| Bad taste, does not like dairy drinks |
| Contains everything you need |
| Contains sugar, not possible with my diabetes |
| Did not notice anything |
| Do not think it works |
| Does not always taste good |
| Doubt whether it is good, but would take it if advised by my physician |
| Fine, good experience with my wife |
| Gains weight using it |
| Gets nauseous, nasty, but I think it would be good for me |
| Good |
| Good protein |
| Good taste (2x) |
| Good taste, good source of vitamins |
| I do not know what it does |
| I do not know, never drank it |
| I do not know, never used, good if physician prescribes it |
| I like it but do not know why I take it |
| I must take it, no specific opinion |
| I take it because I must, but I don't like dairy products |
| If it is not needed, I do not take it |
| If you need it, but do not think it would help me |
| Lots of sugar |
| Nasty |
| Nausea when using it, but I think it is good |
| No experience with it |
| No opinion (5x) |
| No opinion, does not know it |
| No opinion, I use it because given to me |
| No opinion, if good for me then I would take it |
| No opinion, never used |
| No opinion, never used |
| No opinion, probably good for people |
| No specific opinion |
| No understanding of it, would take it if needed |
| Not liking it |
| Not liking jars |
| Not natural, only if really needed |
| Not needed |
| Not needed for now |
| Not needed, no specific opinion |
| Not nice to drink |
| Not real, don't believe it works |
| Only if needed |
| Only if really needed |
| Only if really needed, otherwise better to eat normally |
| Product to get stronger |
| Too milky, do not know whether it contributes to treatment |
| Too sweet (2X) |
| Very bad taste |
| Very good taste |
| Way to earn money |
| Would take if it is good |
