# Supplementary material for: Sarcopenia knowledge of geriatric rehabilitation patients is low while they are willing to start sarcopenia treatment: EMPOWER‐GR
Source: J Cachexia Sarcopenia Muscle. 2023 Dec 20;15(1):352–60. doi: 10.1002/jcsm.13372 (PMC10834324; doi:10.1002/jcsm.13372)
Supplement: Supplementary file 4 — Table S4. Inpatient characteristics that did not complete the sarcopenia survey compared to the total EMPOWER‐GR cohort characteristics. [file JCSM-15-352-s004.docx]

**Table S4.** Inpatient characteristics that did not complete the sarcopenia survey compared to the total EMPOWER-GR cohort characteristics.

| **Characteristics** | **n** | **Included patients**  **(n=157)** | **n** | **Non respondents**  **(n=43)** | ***p*** |
| --- | --- | --- | --- | --- | --- |
| Age (years), mean ± SD | 157 | 80.5 ± 7.3 | 43 | 80.8 ± 7.6 | 0.894 |
| Female, n (%) | 157 | 94 (59.9) | 43 | 19 (44.2) | 0.064 |
| Reason for acute admission, n (%) | 157 |  | 43 |  |  |
| Musculoskeletal |  | 62 (39.5) |  | 11 (25.6) | 0.083 |
| Neurological |  | 28 (17.8) |  | 13 (30.2) | 0.064 |
| Infection |  | 22 (14.0) |  | 8 (18.6) | 0.475 |
| Cardiac |  | 12 (7.6) |  | 1 (2.3) | 0.246 |
| Cancer |  | 11 (7.0) |  | 3 (7.0) | 0.978 |
| Gastrointestinal |  | 10 (6.4) |  | 1 (2.3) | 0.296 |
| Other |  | 12 (7.6) |  | 6 (14.0) | 0.323 |
| CIRS score (0-56) (points) | 157 | 9 [7-13] | 42 | 12 [9-15] | **0.005** |
| Cognitive impairment, n (%) | 157 | 46 (29.3) | 43 | 24 (55.8) | **0.002** |
| Body mass index (kg/m^2^) | 156 | 25.7 [23.1-29.5] | 42 | 23.8 [20.7-27.5] | **0.009** |
| Malnutrition (GLIM), n (%) | 137 | 100 (73.0) | 36 | 30 (83.3) | 0.187 |
| Katz-ADL score (0-6) (points) | 157 | 3 [2-6] | 43 | 2 [1-3] | **0.002** |
| Lawton & Brody-IADL score (0-8) (points) | 154 | 3 [2-4] | 41 | 2 [1-2] | **<0.001** |
| SPPB score (0-12) (points) | 156 | 2 [0-5] | 39 | 0 [0-5] | 0.334 |
| Handgrip strength (kg) | 157 | 17.5 [13.0-24.0] | 41 | 15.0 [10.0-23.0] | 0.385 |
| ALMI (kg/m^2^) | 132 | 6.00 [6.73-7.82] | 34 | 6.83 [5.72-7.70] | 0.887 |
| Sarcopenia (EWGSOP2), n (%) | 138 | 30 (21.7) | 34 | 14 (41.2) | **0.017** |

All data presented as medians [IQR] unless noted otherwise. IQR: interquartile range; SD: standard deviation; CIRS: Cumulative Illness Rating Scale; GLIM: Global Leadership Initiative on Malnutrition; ADL: Activities of Daily Living; SPPB: Short Physical Performance Battery; ALMI: Appendicular Lean Mass Index; EWGSOP2: European Working Group on Sarcopenia in Older People revised definition.
