# Supplementary material for: Sarcopenia knowledge of geriatric rehabilitation patients is low while they are willing to start sarcopenia treatment: EMPOWER‐GR
Source: J Cachexia Sarcopenia Muscle. 2023 Dec 20;15(1):352–60. doi: 10.1002/jcsm.13372 (PMC10834324; doi:10.1002/jcsm.13372)
Supplement: Supplementary file 5 — Table S5. Inpatients' perception of “muscle poverty” (Dutch translation: “spierarmoede”) (n = 40). [file JCSM-15-352-s005.docx]

**Table S5.** Inpatients’ perception of “muscle poverty” (Dutch translation: “spierarmoede”) (n=40).

| **In Dutch** | **English translation** |
| --- | --- |
| Afnemen van spierkracht, massa, functie | Decrease in muscle strength, mass, function |
| Afname spierkracht | Decrease in muscle strength |
| Geen kracht in de armen | No strength in the arms |
| Weinig spieren | Few muscles |
| Het verlies van spiermassa | The loss of muscle mass |
| Sneller spierverlies dan wordt aangemaakt | Faster muscle loss than synthesis |
| Huidige situatie, oftewel oud worden | Current situation, i.e. getting old |
| Moeheid en last van de bloedvaten | Tiredness and problems with blood vessels |
| Vermindering van draagkracht en souplesse | Reduction of bearing capacity and flexibility |
| Verslapping spieren door te weinig beweging | Muscle weakness due to too little movement |
| Slappe spieren | Weak muscles |
| Weinig kracht | Low strength |
| Te weinig spiermassa | Too little muscle mass |
| Geen kracht | No strength |
| Spieren die niet functioneren | Muscles that do not function |
| Niet getrainde spieren atrofiëren | Untrained muscle atrophy |
| Slappe spieren | Weak muscles |
| Weinig kracht, tillen/bewegen | Low strength, lifting/moving |
| Gauw moe | Very tired |
| Niet genoeg spieren | Not enough muscles |
| Spieren die afsterven, niet genoeg kracht | Muscles dying, not enough strength |
| Spierdystrofie | Muscular dystrophy |
| Slappe spieren | Weak muscles |
| Niet veel kracht | Not much strength |
| Spieren niet goed doen, onwillig | Muscles not working well, unwilling |
| Spiermassa verlies | Muscle mass loss |
| Verlamming van de spieren | Muscle paralysis |
| Afname spierkracht (verzuring) | Decrease in muscle strength (acidification) |
| Slappe spieren | Weak muscles |
| Weinig kracht | Low strength |
| Minder spieren | Less muscles |
| Spier kwijt door op bed te liggen | Muscle loss due to lying in bed |
| Spieren niet goed kan gebruiken | Unable to use muscles well |
| Minder krachtige spieren | Less strength in muscles |
| Tekort aan spieren | Muscle shortage |
| Als je te slap wordt | When you are becoming too weak |
| Spierziekte bij niet genoeg bewegen | Muscle disease due to too little movement |
| Spierziekte | Muscle disease |
| Als de spieren het niet doen | When the muscles are not working |
| Aan spieren werken om het goed te krijgen | Working on muscles to get better |
