# Supplementary material for: Sarcopenia knowledge of geriatric rehabilitation patients is low while they are willing to start sarcopenia treatment: EMPOWER‐GR
Source: J Cachexia Sarcopenia Muscle. 2023 Dec 20;15(1):352–60. doi: 10.1002/jcsm.13372 (PMC10834324; doi:10.1002/jcsm.13372)
Supplement: Supplementary file 6 — Table S6. A. Survey answers stratified by sarcopenia status, musculoskeletal condition and cognitive impairment. B. Survey answers stratified by professional background, education and living situation. [file JCSM-15-352-s006.docx]

**Table S6A.** Survey answers stratified by sarcopenia status, **musculoskeletal condition and cognitive impairment**.

|  | **Sarcopenia** | | | | |  | **Musculoskeletal condition** | | | | |  | **Cognitive impairment** | | | | |
| --- | --- | --- | --- | --- | --- | --- | --- | --- | --- | --- | --- | --- | --- | --- | --- | --- | --- |
|  | **n** | **Yes**^a^ | **n** | **No** | ***p*** |  | **n** | **Yes** | **n** | **No** | ***p*** |  | **n** | **Yes** | **n** | **No** | ***p*** |
| **Sarcopenia knowledge** |  |  |  |  |  |  |  |  |  |  |  |  |  |  |  |  |  |
| Heard of term sarcopenia | 100 | 2 (2.0) | 57 | 3 (5.3) | ^b^ |  | 120 | 4 (3.3) | 37 | 1 (2.7) | ^b^ |  | 46 | 0 (0.0) | 111 | 5 (4.5) | ^b^ |
| Knows what sarcopenia is | 100 | 2 (2.0) | 57 | 3 (5.3) | ^b^ |  | 120 | 4 (3.3) | 37 | 1 (2.7) | ^b^ |  | 46 | 0 (0.0) | 111 | 5 (4.5) | ^b^ |
| Identifies sarcopenia as a disease of | 98 |  | 52 |  | 0.082 |  | 115 |  | 35 |  | 0.564 |  | 43 |  | 107 |  | 0.339 |
| Muscle tissue |  | 5 (5.1) |  | 8 (15.4) |  |  |  | 11 (9.6) |  | 2 (5.7) |  |  |  | 1 (2.3) |  | 12 (11.2) |  |
| Brain tissue |  | 2 (2.0) |  | 1 (1.9) |  |  |  | 2 (1.7) |  | 1 (2.9) |  |  |  | 1 (2.3) |  | 2 (1.9) |  |
| Other (fat tissue, heart, bones, joints) |  | 1 (1.0) |  | 3 (5.8) |  |  |  | 3 (2.6) |  | 1 (2.9) |  |  |  | 1 (2.3) |  | 3 (2.8) |  |
| Don't know |  | 90 (91.8) |  | 40 (76.9) |  |  |  | 99 (86.1) |  | 31 (88.6) |  |  |  | 40 (93.0) |  | 90 (84.1) |  |
| Heard of term "muscle poverty" | 100 | 26 (26.0) | 57 | 14 (24.6) | 0.842 |  | 120 | 32 (26.7) | 37 | 8 (21.6) | 0.538 |  | 46 | 12 (26.1) | 111 | 28 (25.2) | 0.910 |
| Nutrients important for muscle health ^c^ | 94 |  | 54 |  |  |  | 115 |  | 37 |  |  |  | 41 |  | 107 |  |  |
| Protein |  | 53 (56.4) |  | 41 (75.9) | **0.017** |  |  | 73 (63.5) |  | 21 (63.6) | 0.987 |  |  | 17 (41.5) |  | 77 (72.0) | **<.001** |
| Sugar |  | 8 (8.5) |  | 5 (9.3) | 0.742 |  |  | 10 (8.7) |  | 3 (9.1) | ^b^ |  |  | 4 (9.8) |  | 9 (8.4) | ^b^ |
| Fat |  | 8 (8.5) |  | 5 (9.3) | 0.877 |  |  | 10 (8.7) |  | 3 (9.1) | ^b^ |  |  | 5 (12.2) |  | 8 (7.5) | 0.364 |
| Energy |  | 16 (17.0) |  | 10 (18.5) | 0.818 |  |  | 21 (18.3) |  | 5 (15.2) | 0.679 |  |  | 7 (17.1) |  | 19 (17.8) | 0.922 |
| Vitamins |  | 31 (33.0) |  | 23 (42.6) | 0.242 |  |  | 44 (38.3) |  | 10 (30.3) | 0.403 |  |  | 12 (29.3) |  | 42 (39.3) | 0.259 |
| Minerals |  | 13 (13.8) |  | 15 (27.8) | **0.037** |  |  | 20 (17.4) |  | 8 (24.2) | 0.376 |  |  | 7 (17.1) |  | 21 (19.6) | 0.723 |
| Don't know |  | 27 (28.7) |  | 9 (16.7) | 0.100 |  |  | 28 (24.3) |  | 8 (24.2) | 0.990 |  |  | 15 (36.6) |  | 21 (19.6) | **0.031** |
| Age when muscle begins to decline (years) | 73 | 60 [50-70] | 47 | 60 [50-70] | 0.820 |  | 95 | 60 [50-70] | 25 | 60 [48-70] | 0.878 |  |  | 60 [50-77] |  | 60 [50-65] | 0.093 |
| Prevalence of sarcopenia in GR inpatients | 93 |  | 54 |  | 0.716 |  | 114 |  | 33 |  | 0.273 |  | 40 |  | 107 |  | 0.711 |
| <10% |  | 1 (1.1) |  | 2 (3.7) |  |  |  | 1 (0.9) |  | 2 (6.1) |  |  |  | 1 (2.5) |  | 2 (1.9) |  |
| 10-20% |  | 0 (0.0) |  | 0 (0.0) |  |  |  | 0 (0.0) |  | 0 (0.0) |  |  |  | 0 (0.0) |  | 0 (0.0) |  |
| 20-30% |  | 1 (1.1) |  | 0 (0.0) |  |  |  | 1 (0.9) |  | 0 (0.0) |  |  |  | 1 (2.5) |  | 0 (0.0) |  |
| 30-40% |  | 8 (8.6) |  | 7 (13.0) |  |  |  | 10 (8.8) |  | 5 (15.2) |  |  |  | 4 (10.0) |  | 11 (10.3) |  |
| 40-50% |  | 9 (9.7) |  | 4 (7.4) |  |  |  | 12 (10.5) |  | 1 (3.0) |  |  |  | 4 (10.0) |  | 9 (8.4) |  |
| >50% |  | 27 (29.0) |  | 13 (24.1) |  |  |  | 31 (27.2) |  | 9 (27.3) |  |  |  | 10 (25.0) |  | 30 (28.0) |  |
| Don't know |  | 47 (50.5) |  | 28 (51.9) |  |  |  | 59 (51.8) |  | 16 (48.5) |  |  |  | 20 (50.0) |  | 55 (51.4) |  |
| **Willingness and barriers to sarcopenia treatment** | |  |  |  |  |  |  |  |  |  |  |  |  |  |  |  |  |
| Willing to start treatment if diagnosed |  |  |  |  |  |  |  |  |  |  |  |  |  |  |  |  |  |
| Before explaining what treatment is | 92 | 75 (81.5) | 54 | 50 (92.6) | 0.066 |  | 114 | 98 (86.0) | 32 | 27 (84.4) | 0.821 |  | 40 | 33 (82.5) | 106 | 92 (86.8) | 0.510 |
| After explaining what treatment is^c^ | 95 |  | 54 |  |  |  |  |  |  |  |  |  | 40 |  | 109 |  |  |
| RET 3x/week for 3 months |  | 62 (65.3) |  | 38 (70.4) | 0.524 |  |  | 82 (71.3) |  | 18 (52.9) | **0.045** |  |  | 24 (60.0) |  | 76 (69.7) | 0.263 |
| ONS 2x/day for 3 months |  | 56 (58.9) |  | 27 (50.0) | 0.291 |  |  | 67 (58.3) |  | 16 (47.1) | 0.248 |  |  | 24 (60.0) |  | 59 (54.1) | 0.523 |
| High-protein diet on a daily basis |  | 49 (51.6) |  | 42 (77.8) | **0.002** |  |  | 74 (64.3) |  | 17 (50.0) | 0.132 |  |  | 20 (50.0) |  | 71 (65.1) | 0.093 |
| None |  | 12 (12.6) |  | 3 (5.6) | ^b^ |  |  | 9 (7.8) |  | 6 (17.6) | 0.095 |  |  | 6 (15.0) |  | 9 (8.3) | 0.225 |
| Willing and able to take part in RET now | 95 | 53 (55.5) | 54 | 34 (63.0) | 0.393 |  | 115 | 71 (61.7) | 34 | 16 (47.1) | 0.127 |  | 41 | 21 (51.2) | 108 | 66 (61.1) | 0.274 |
| If no, why?^c^ | 42 |  | 20 |  |  |  | 44 |  | 18 |  |  |  | 20 |  | 42 |  |  |
| Too intensive |  | 11 (26.2) |  | 9 (45.0) | 0.139 |  |  | 13 (29.5) |  | 7 (38.9) | 0.475 |  |  | 7 (35.0) |  | 13 (31.0) | 0.750 |
| Too difficult |  | 8 (19.0) |  | 2 (10.0) | ^b^ |  |  | 8 (18.2) |  | 2 (11.1) | ^b^ |  |  | 4 (20.0) |  | 6 (14.3) | ^b^ |
| Could be harmful |  | 2 (4.8) |  | 0 (0.0) | ^b^ |  |  | 2 (4.5) |  | 0 (0.0) | ^b^ |  |  | 1 (5.0) |  | 1 (2.4) | ^b^ |
| Other |  | 26 (61.9) |  | 13 (65.0) | 0.814 |  |  | 29 (65.9) |  | 10 (55.6) | 0.444 |  |  | 12 (60.0) |  | 27 (64.3) | 0.744 |
| Not motivated |  | 8 (19.0) |  | 0 (0.0) | ^b^ |  |  | 4 (9.1) |  | 4 (22.2) | ^b^ |  |  | 2 (10.0) |  | 6 (14.3) | ^b^ |
| Not important/ not necessary |  | 7 (16.7) |  | 4 (20.0) | ^b^ |  |  | 8 (18.2) |  | 3 (16.7) | ^b^ |  |  | 3 (15.0) |  | 8 (19.0) | ^b^ |
| Health issues/ too tired |  | 7 (16.7) |  | 6 (30.0) | 0.228 |  |  | 12 (27.3) |  | 1 (5.6) | ^b^ |  |  | 6 (30.0) |  | 7 (16.7) | 0.228 |
| Barriers to sarcopenia treatment^c^ | 93 |  | 54 |  |  |  | 113 |  | 34 |  |  |  | 40 |  | 107 |  |  |
| Takes too much time |  | 2 (2.2) |  | 4 (7.4) | ^b^ |  |  | 5 (4.4) |  | 1 (2.9) | ^b^ |  |  | 1 (2.5) |  | 5 (4.7) | ^b^ |
| Too many other health issues |  | 12 (12.9) |  | 8 (14.8) | 0.745 |  |  | 16 (14.2) |  | 4 (11.8) | ^b^ |  |  | 5 (12.5) |  | 15 (14.0) | 0.811 |
| Dislike ONS |  | 14 (15.1) |  | 11 (20.4) | 0.408 |  |  | 18 (15.9) |  | 7 (20.6) | 0.526 |  |  | 5 (12.5) |  | 20 (18.7) | 0.374 |
| Dislike physical activity |  | 5 (5.4) |  | 4 (7.4) | ^b^ |  |  | 5 (4.4) |  | 4 (11.8) | ^b^ |  |  | 0 (0.0) |  | 9 (8.4) | ^b^ |
| No supervision for RET when going home |  | 7 (7.5) |  | 2 (3.7) | ^b^ |  |  | 6 (5.3) |  | 3 (8.8) | ^b^ |  |  | 2 (5.0) |  | 7 (6.5) | ^b^ |
| HCP too far away after discharge |  | 1 (1.1) |  | 0 (0.0) | ^b^ |  |  | 1 (0.9) |  | 0 (0.0) | ^b^ |  |  | 1 (2.5) |  | 0 (0.0) | ^b^ |
| Treatment expensive |  | 4 (4.3) |  | 4 (7.4) | ^b^ |  |  | 4 (3.5) |  | 4 (11.8) | ^b^ |  |  | 2 (5.0) |  | 6 (5.6) | ^b^ |
| Consequences not severe enough |  | 1 (1.1) |  | 1 (1.9) | ^b^ |  |  | 2 (1.8) |  | 0 (0.0) | ^b^ |  |  | 0 (0.0) |  | 2 (1.9) | ^b^ |
| Dislike to go to healthcare provider |  | 0 (0.0) |  | 2 (3.7) | ^b^ |  |  | 1 (0.9) |  | 1 (2.9) | ^b^ |  |  | 0 (0.0) |  | 2 (1.9) | ^b^ |
| Other |  | 56 (60.2) |  | 21 (38.9) | **0.013** |  |  | 61 (54.0) |  | 16 (47.1) | 0.479 |  |  | 25 (62.5) |  | 52 (48.6) | 0.133 |
| Dislike/difficulty to adjust diet |  | 11 (11.8) |  | 5 (9.3) | 0.630 |  |  | 13 (11.5) |  | 3 (8.8) | ^b^ |  |  | 4 (10.0) |  | 12 (11.2) | ^b^ |
| Dislike RET specifically |  | 3 (3.2) |  | 1 (1.9) | ^b^ |  |  | 3 (2.7) |  | 1 (2.9) | ^b^ |  |  | 1 (2.5) |  | 3 (2.8) | ^b^ |
| RET too intense/difficult |  | 14 (15.1) |  | 1 (1.9) | ^b^ |  |  | 10 (8.8) |  | 5 (14.7) | 0.323 |  |  | 5 (12.5) |  | 10 (9.3) | 0.574 |
| Dislike/difficulty to leave home |  | 3 (3.2) |  | 2 (3.7) | ^b^ |  |  | 5 (4.4) |  | 0 (0.0) | ^b^ |  |  | 3 (7.5) |  | 2 (1.9) | ^b^ |
| Doubts about treatment effectiveness |  | 14 (15.1) |  | 5 (9.3) | 0.313 |  |  | 13 (11.5) |  | 6 (17.6) | 0.349 |  |  | 6 (15.0) |  | 13 (12.1) | 0.647 |
| No barriers |  | 18 (19.4) |  | 16 (29.6) | 0.154 |  |  | 26 (23.0) |  | 8 (23.5) | 0.950 |  |  | 8 (20.0) |  | 26 (24.3) | 0.582 |
| Taking ONS now or in the past |  | 46 (49.5) |  | 16 (29.6) | **0.019** |  |  | 48 (42.1) |  | 14 (42.4) | 0.974 |  | 41 | 19 (46.3) | 106 | 43 (40.6) | 0.525 |
| Opinion on ONS^c^ | 93 |  | 52 |  |  |  | 115 |  | 34 |  |  |  | 39 |  | 106 |  |  |
| Useful to increase nutritional intake |  | 10 (10.8) |  | 4 (7.7) | ^b^ |  |  | 11 (9.8) |  | 3 (9.1) | ^b^ |  |  | 7 (17.9) |  | 7 (6.6) | **0.040** |
| Good protein source |  | 15 (16.1) |  | 9 (17.3) | 0.855 |  |  | 17 (15.2) |  | 7 (21.2) | 0.412 |  |  | 5 (12.8) |  | 19 (17.9) | 0.463 |
| High in calories |  | 1 (1.1) |  | 4 (7.7) | ^b^ |  |  | 3 (2.7) |  | 2 (6.1) | ^b^ |  |  | 1 (2.6) |  | 4 (3.8) | ^b^ |
| Contributes to treatment |  | 29 (31.2) |  | 12 (23.1) | 0.299 |  |  | 32 (28.6) |  | 9 (27.3) | 0.884 |  |  | 11 (28.2) |  | 30 (28.3) | 0.991 |
| Extra medication |  | 4 (4.3) |  | 1 (1.9) | ^b^ |  |  | 5 (4.5) |  | 0 (0.0) | ^b^ |  |  | 2 (5.1) |  | 3 (2.8) | ^b^ |
| Does not contribute to treatment |  | 6 (6.5) |  | 3 (5.8) | ^b^ |  |  | 7 (6.3) |  | 2 (6.1) | ^b^ |  |  | 3 (7.7) |  | 6 (5.7) | ^b^ |
| Do not know what ONS is |  | 15 (16.1) |  | 12 (23.1) | 0.303 |  |  | 23 (20.5) |  | 4 (12.1) | ^b^ |  |  | 9 (23.1) |  | 18 (17.0) | 0.403 |
| Other |  | 46 (49.5) |  | 19 (36.5) | 0.133 |  |  | 50 (44.6) |  | 15 (45.5) | 0.934 |  |  | 15 (38.5) |  | 50 (47.2) | 0.350 |
| Does not taste good/ too sweet |  | 15 (16.1) |  | 6 (11.5) | 0.451 |  |  | 18 (16.1) |  | 3 (9.1) | ^b^ |  |  | 5 (12.8) |  | 16 (15.1) | 0.730 |
| No opinion |  | 15 (16.1) |  | 6 (11.5) | 0.451 |  |  | 16 (14.3) |  | 5 (15.2) | 0.901 |  |  | 3 (7.7) |  | 18 (17.0) | ^b^ |
| Willing to prevent sarcopenia^c^ | 91 |  | 54 |  |  |  | 112 |  | 33 |  |  |  | 39 |  | 106 |  |  |
| Yes, increase physical activity |  | 53 (58.2) |  | 30 (55.6) | 0.752 |  |  | 69 (61.6) |  | 14 (42.4) | 0.050 |  |  | 21 (53.8) |  | 62 (58.5) | 0.616 |
| Yes, increase activities increasing strength |  | 44 (48.4) |  | 32 (59.4) | 0.204 |  |  | 64 (57.1) |  | 12 (36.4) | **0.036** |  |  | 20 (51.3) |  | 56 (52.8) | 0.869 |
| Yes, increase protein intake |  | 47 (51.6) |  | 30 (55.6) | 0.649 |  |  | 63 (56.3) |  | 14 (42.4) | 0.162 |  |  | 16 (41.0) |  | 61 (57.5) | 0.077 |
| No |  | 28 (30.8) |  | 15 (27.8) | 0.703 |  |  | 31 (27.7) |  | 12 (36.4) | 0.337 |  |  | 15 (38.5) |  | 28 (26.4) | 0.159 |

All values are reported as n (%) or median [IQR]. ^a^ Including patients with probable and confirmed sarcopenia. ^b^ P-value not shown because of sample size below five. ^c^ Multiple answers possible. IQR: interquartile range; GR: geriatric rehabilitation; RET: resistance exercise training; ONS: oral nutritional supplement.

**Table S6B. Survey answers stratified by professional background, education and living situation.**

|  | **Professional background** | | | | |  | **Education** | | | | |  | **Living Situation** | | | | |
| --- | --- | --- | --- | --- | --- | --- | --- | --- | --- | --- | --- | --- | --- | --- | --- | --- | --- |
|  | **n** | **Health** | **n** | **Other** | ***p*** |  | **n** | **Higher** | **n** | **Lower** | ***p*** |  | **n** | **Alone** | **n** | **Other** | ***p*** |
| **Sarcopenia knowledge** |  |  |  |  |  |  |  |  |  |  |  |  |  |  |  |  |  |
| Heard of term sarcopenia | 11 | 1 (9.1) | 139 | 4 (2.9) | ^b^ |  | 49 | 2 (4.1) | 105 | 3 (2.9) | ^b^ |  | 109 | 3 (2.8) | 46 | 2 (4.3) | ^b^ |
| Knows what sarcopenia is | 11 | 1 (9.1) | 139 | 4 (2.9) | ^b^ |  | 49 | 2 (4.1) | 105 | 3 (2.9) | ^b^ |  | 109 | 3 (2.8) | 46 | 2 (4.3) | ^b^ |
| Identifies sarcopenia as a disease of | 10 |  | 135 |  | 0.997 |  | 46 |  | 101 |  | 0.542 |  | 104 |  | 44 |  | 0.654 |
| Muscle tissue |  | 1 (10.0) |  | 12 (8.9) |  |  |  | 6 (13.0) |  | 7 (6.9) |  |  |  | 8 (7.7) |  | 5 (11.4) |  |
| Brain tissue |  | 0 (0.0) |  | 3 (2.2) |  |  |  | 2 (4.3) |  | 1 (1.0) |  |  |  | 1 (1.0) |  | 2 (4.5) |  |
| Other (fat tissue, heart, bones, joints) |  | 0 (0.0) |  | 4 (3.0) |  |  |  | 0 (0.0) |  | 4 (4.0) |  |  |  | 4 (3.8) |  | 0 (0.0) |  |
| Don't know |  | 9 (90.0) |  | 116 (85.9) |  |  |  | 38 (82.6) |  | 89 (88.1) |  |  |  | 91 (87.5) |  | 37 (84.1) |  |
| Heard of term "muscle poverty" | 11 | 1 (9.1) | 139 | 38 (27.3) | ^b^ |  | 49 | 14 (28.6) | 105 | 25 (23.8) | 0.527 |  | 109 | 13 (28.3) | 46 | 27 (24.8) | 0.650 |
| Nutrients important for muscle health | 11 |  | 132 |  |  |  | 44 |  | 102 |  |  |  | 104 |  | 42 |  |  |
| Protein |  | 10 (90.9) |  | 80 (60.6) | **0.046** |  |  | 34 (77.3) |  | 60 (58.8) | **0.033** |  |  | 64 (61.5) |  | 28 (66.7) | 0.561 |
| Sugar |  | 0 (0.0) |  | 13 (9.8) | ^b^ |  |  | 4 (9.1) |  | 8 (7.8) | ^b^ |  |  | 10 (9.6) |  | 3 (7.1) | ^b^ |
| Fat |  | 0 (0.0) |  | 11 (8.3) | ^b^ |  |  | 3 (6.8) |  | 9 (8.8) | ^b^ |  |  | 10 (9.6) |  | 3 (7.1) | ^b^ |
| Energy |  | 1 (9.1) |  | 24 (18.2) | ^b^ |  |  | 8 (18.2) |  | 18 (17.6) | 0.938 |  |  | 18 (17.3) |  | 7 (16.7) | 0.926 |
| Vitamins |  | 2 (18.2) |  | 48 (36.4) | ^b^ |  |  | 21 (47.7) |  | 32 (31.4) | 0.059 |  |  | 40 (38.5) |  | 13 (31.0) | 0.393 |
| Minerals |  | 2 (18.2) |  | 24 (18.2) | ^b^ |  |  | 14 (31.8) |  | 14 (13.7) | **0.011** |  |  | 19 (18.3) |  | 8 (19.0) | 0.913 |
| Don't know |  | 1 (9.1) |  | 35 (26.5) | ^b^ |  |  | 7 (15.9) |  | 29 (28.4) | 0.107 |  |  | 26 (25.0) |  | 10 (23.8) | 0.880 |
| Age when muscle begins to decline (years) | 11 | 50 [45-75] | 105 | 60 [50-70] | 0.497 |  | 41 | 60 [48-66] | 78 | 60 [50-70] | 0.542 |  | 84 | 60 [50-70] | 35 | 65 [50-75] | 0.302 |
| Prevalence of sarcopenia in GR inpatients | 11 |  | 131 |  | 0.528 |  | 44 |  | 101 |  | 0.266 |  | 103 |  | 42 |  | 0.659 |
| <10% |  | 0 (0.0) |  | 3 (2.3) |  |  |  | 0 (0.0) |  | 3 (3.0) |  |  |  | 2 (1.9) |  | 1 (2.4) |  |
| 10-20% |  | 0 (0.0) |  | 0 (0.0) |  |  |  | 0 (0.0) |  | 0 (0.0) |  |  |  | 0 (0.0) |  | 0 (0.0) |  |
| 20-30% |  | 0 (0.0) |  | 1 (0.8) |  |  |  | 1 (2.3) |  | 0 (0.0) |  |  |  | 1 (1.0) |  | 0 (0.0) |  |
| 30-40% |  | 1 (9.1) |  | 12 (9.2) |  |  |  | 7 (15.9) |  | 8 (7.9) |  |  |  | 9 (8.7) |  | 6 (14.3) |  |
| 40-50% |  | 2 (18.2) |  | 11 (8.4) |  |  |  | 5 (11.4) |  | 8 (7.9) |  |  |  | 8 (7.8) |  | 5 (11.9) |  |
| >50% |  | 5 (45.5) |  | 34 (26.0) |  |  |  | 10 (22.7) |  | 29 (28.7) |  |  |  | 31 (30.1) |  | 8 (19.0) |  |
| Don't know |  | 3 (27.3) |  | 70 (53.4) |  |  |  | 21 (47.7) |  | 53 (52.5) |  |  |  | 52 (50.5) |  | 22 (52.4) |  |
| **Willingness and barriers to sarcopenia treatment** | | |  |  |  |  |  |  |  |  |  |  |  |  |  |  |  |
| Willing to start treatment if diagnosed |  |  |  |  |  |  |  |  |  |  |  |  |  |  |  |  |  |
| Before explaining what treatment is | 11 | 10 (90.9) | 130 | 110 (84.6) | 0.573 |  | 43 | 40 (93.0) | 100 | 82 (82.0) | 0.088 |  | 103 | 87 (84.5) | 41 | 36 (87.8) | 0.608 |
| After explaining what treatment is^c^ | 11 |  | 132 |  |  |  | 45 |  | 101 |  |  |  | 105 |  | 42 |  |  |
| RET 3x/week for 3 months |  | 6 (54.5) |  | 92 (69.7) | 0.299 |  |  | 32 (71.1) |  | 68 (67.3) | 0.649 |  |  | 70 (66.7) |  | 28 (66.7) | 1.000 |
| ONS 2x/day for 3 months |  | 7 (63.6) |  | 74 (56.1) | 0.626 |  |  | 25 (55.6) |  | 57 (56.4) | 0.921 |  |  | 59 (56.2) |  | 22 (52.4) | 0.675 |
| High-protein diet on a daily basis |  | 10 (90.9) |  | 76 (57.6) | **0.030** |  |  | 30 (66.7) |  | 59 (58.4) | 0.345 |  |  | 67 (63.8) |  | 23 (54.8) | 0.309 |
| None |  | 0 (0.0) |  | 15 (11.4) | ^b^ |  |  | 3 (6.7) |  | 12 (11.9) | ^b^ |  |  | 11 (10.5) |  | 4 (9.5) | ^b^ |
| Willing and able to take part in RET now | 11 | 6 (54.5) | 132 | 78 (59.1) | 0.769 |  | 46 | 27 (58.7) | 100 | 60 (60.0) | 0.881 |  | 104 | 62 (59.6) | 43 | 24 (55.8) | 0.670 |
| If no, why?^c^ | 5 |  | 54 |  |  |  | 19 |  | 40 |  |  |  | 42 |  | 19 |  |  |
| Too intensive |  | 4 (80.0) |  | 16 (29.6) | ^b^ |  |  | 7 (36.8) |  | 12 (30.0) | 0.599 |  |  | 15 (35.7) |  | 5 (26.3) | 0.469 |
| Too difficult |  | 1 (20.0) |  | 9 (16.7) | ^b^ |  |  | 1 (5.3) |  | 8 (20.0) | ^b^ |  |  | 7 (16.7) |  | 3 (15.8) | ^b^ |
| Could be harmful |  | 0 (0.0) |  | 1 (1.9) | ^b^ |  |  | 0 (0.0) |  | 2 (5.0) | ^b^ |  |  | 1 (2.4) |  | 0 (0.0) | ^b^ |
| Other |  | 2 (40.0) |  | 35 (64.8) | ^b^ |  |  | 12 (63.2) |  | 24 (60.0) | 0.816 |  |  | 25 (59.5) |  | 13 (68.4) | 0.507 |
| Not motivated |  | 0 (0.0) |  | 8 (14.8) | ^b^ |  |  | 3 (15.8) |  | 5 (12.5) | ^b^ |  |  | 6 (14.3) |  | 2 (10.5) | ^b^ |
| Not important/ not necessary |  | 1 (20.0) |  | 10 (18.5) | ^b^ |  |  | 2 (10.5) |  | 9 (22.5) | ^b^ |  |  | 6 (14.3) |  | 5 (26.3) | 0.258 |
| Health issues/ too tired |  | 0 (0.0) |  | 11 (20.4) | ^b^ |  |  | 1 (5.3) |  | 9 (22.5) | ^b^ |  |  | 9 (21.4) |  | 3 (15.8) | ^b^ |
| Barriers to sarcopenia treatment^c^ | 11 |  | 130 |  |  |  | 44 |  | 100 |  |  |  | 104 |  | 41 |  |  |
| Takes too much time |  | 0 (0.0) |  | 6 (4.6) | ^b^ |  |  | 1 (2.3) |  | 5 (5.0) | ^b^ |  |  | 4 (3.8) |  | 2 (4.9) | ^b^ |
| Too many other health issues |  | 2 (18.2) |  | 16 (12.3) | ^b^ |  |  | 4 (9.1) |  | 14 (14.0) | ^b^ |  |  | 14 (13.5) |  | 5 (12.2) | 0.839 |
| Dislike ONS |  | 1 (9.1) |  | 23 (17.7) | ^b^ |  |  | 6 (13.6) |  | 18 (18.0) | 0.517 |  |  | 21 (20.2) |  | 4 (9.8) | ^b^ |
| Dislike physical activity |  | 2 (18.2) |  | 6 (4.6) | ^b^ |  |  | 3 (6.8) |  | 5 (5.0) | ^b^ |  |  | 5 (4.8) |  | 4 (9.8) | ^b^ |
| No supervision for RET when going home |  | 0 (0.0) |  | 8 (6.2) | ^b^ |  |  | 4 (9.1) |  | 5 (5.0) | ^b^ |  |  | 6 (5.8) |  | 3 (7.3) | ^b^ |
| HCP too far away after discharge |  | 0 (0.0) |  | 1 (0.8) | ^b^ |  |  | 1 (2.3) |  | 0 (0.0) | ^b^ |  |  | 0 (0.0) |  | 1 (2.4) | ^b^ |
| Treatment expensive |  | 0 (0.0) |  | 7 (5.4) | ^b^ |  |  | 1 (2.3) |  | 7 (7.0) | ^b^ |  |  | 4 (3.8) |  | 3 (7.3) | ^b^ |
| Consequences not severe enough |  | 0 (0.0) |  | 2 (1.5) | ^b^ |  |  | 1 (2.3) |  | 1 (1.0) | ^b^ |  |  | 1 (1.0) |  | 1 (2.4) | ^b^ |
| Dislike to go to healthcare provider |  | 0 (0.0) |  | 2 (1.5) | ^b^ |  |  | 0 (0.0) |  | 2 (2.0) | ^b^ |  |  | 2 (1.9) |  | 0 (0.0) | ^b^ |
| Other |  | 7 (63.6) |  | 68 (52.3) | 0.470 |  |  | 23 (52.3) |  | 52 (52.0) | 0.976 |  |  | 59 (56.7) |  | 17 (41.5) | 0.097 |
| Dislike/difficulty to adjust diet |  | 0 (0.0) |  | 16 (12.3) | ^b^ |  |  | 3 (6.8) |  | 13 (13.0) | ^b^ |  |  | 15 (14.4) |  | 0 (0.0) | ^b^ |
| Dislike RET specifically |  | 0 (0.0) |  | 4 (3.1) | ^b^ |  |  | 2 (4.5) |  | 2 (2.0) | ^b^ |  |  | 3 (2.9) |  | 0 (0.0) | ^b^ |
| RET too intense/difficult |  | 2 (18.2) |  | 12 (9.2) | ^b^ |  |  | 1 (2.3) |  | 12 (12.0) | ^b^ |  |  | 13 (12.5) |  | 0 (0.0) | ^b^ |
| Dislike/difficulty to leave home |  | 1 (9.1) |  | 4 (3.1) | ^b^ |  |  | 3 (6.8) |  | 2 (2.0) | ^b^ |  |  | 2 (1.9) |  | 3 (7.3) | ^b^ |
| Doubts about treatment effectiveness |  | 2 (18.2) |  | 16 (12.3) | ^b^ |  |  | 5 (11.4) |  | 14 (14.0) | 0.667 |  |  | 14 (13.5) |  | 5 (12.2) | 0.839 |
| No barriers |  | 2 (18.2) |  | 31 (23.8) | ^b^ |  |  | 11 (25.0) |  | 23 (23.0) | 0.795 |  |  | 24 (23.1) |  | 10 (24.4) | 0.867 |
| Taking ONS now or in the past | 11 | 5 (45.5) | 132 | 57 (43.2) | 0.884 |  | 46 | 21 (45.7) | 99 | 40 (40.4) | 0.551 |  | 102 | 40 (39.2) | 43 | 21 (48.8) | 0.284 |
| Opinion on ONS^c^ | 10 |  | 130 |  |  |  | 45 |  | 99 |  |  |  | 100 |  | 43 |  |  |
| Useful to increase nutritional intake |  | 1 (10.0) |  | 13 (10.0) | ^b^ |  |  | 4 (8.9) |  | 10 (10.1) | ^b^ |  |  | 7 (7.0) |  | 7 (16.3) | 0.087 |
| Good protein source |  | 3 (30.0) |  | 21 (16.2) | ^b^ |  |  | 11 (24.4) |  | 13 (13.1) | 0.091 |  |  | 14 (14.0) |  | 10 (23.3) | 0.174 |
| High in calories |  | 1 (10.0) |  | 4 (3.1) | ^b^ |  |  | 2 (4.4) |  | 3 (3.0) | ^b^ |  |  | 3 (3.0) |  | 2 (4.7) | ^b^ |
| Contributes to treatment |  | 4 (40.0) |  | 37 (28.5) | ^b^ |  |  | 16 (35.6) |  | 25 (25.3) | 0.204 |  |  | 26 (26.0) |  | 14 (32.6) | 0.423 |
| Extra medication |  | 0 (0.0) |  | 5 (3.8) | ^b^ |  |  | 3 (6.7) |  | 2 (2.0) | ^b^ |  |  | 2 (2.0) |  | 3 (7.0) | ^b^ |
| Does not contribute to treatment |  | 1 (10.0) |  | 7 (5.4) | ^b^ |  |  | 1 (2.2) |  | 7 (7.1) | ^b^ |  |  | 9 (9.0) |  | 0 (0.0) | ^b^ |
| Do not know what ONS is |  | 1 (10.0) |  | 23 (17.7) | ^b^ |  |  | 10 (22.2) |  | 17 (17.2) | 0.472 |  |  | 19 (19.0) |  | 7 (16.3) | 0.699 |
| Other |  | 6 (60.0) |  | 57 (43.8) | 0.322 |  |  | 18 (40.0) |  | 46 (46.5) | 0.469 |  |  | 47 (47.0) |  | 17 (39.5) | 0.410 |
| Does not taste good/ too sweet |  | 3 (30.0) |  | 18 (13.8) | ^b^ |  |  | 8 (17.8) |  | 13 (13.1) | 0.464 |  |  | 18 (18.0) |  | 2 (4.7) | ^b^ |
| No opinion |  | 1 (10.0) |  | 19 (14.6) | ^b^ |  |  | 5 (11.1) |  | 16 (15.2) | 0.426 |  |  | 15 (15.0) |  | 6 (14.0) | 0.871 |
| Willing to prevent sarcopenia^c^ | 11 |  | 129 |  |  |  | 44 |  | 99 |  |  |  | 102 |  | 41 |  |  |
| Yes, increase physical activity |  | 7 (63.6) |  | 73 (56.6) | 0.650 |  |  | 30 (68.2) |  | 52 (52.5) | 0.081 |  |  | 57 (55.9) |  | 24 (58.5) | 0.772 |
| Yes, increase activities increasing strength |  | 7 (63.6) |  | 68 (52.7) | 0.486 |  |  | 28 (63.6) |  | 48 (48.5) | 0.094 |  |  | 57 (55.9) |  | 17 (41.5) | 0.119 |
| Yes, increase protein intake |  | 7 (63.6) |  | 68 (52.7) | 0.486 |  |  | 26 (59.1) |  | 50 (50.5) | 0.342 |  |  | 53 (52.0) |  | 22 (53.7) | 0.854 |
| No |  | 1 (9.1) |  | 41 (31.8) | ^b^ |  |  | 7 (15.9) |  | 35 (35.4) | **0.018** |  |  | 31 (30.4) |  | 12 (29.3) | 0.895 |

All values are reported as n (%) or median [IQR]. ^a^ Including patients with probable and confirmed sarcopenia. ^b^ P-value not shown because of sample size below five. ^c^ Multiple answers possible. IQR: interquartile range; GR: geriatric rehabilitation; RET: resistance exercise training; ONS: oral nutritional supplement.
