# Supplementary material for: Sarcopenia knowledge of geriatric rehabilitation patients is low while they are willing to start sarcopenia treatment: EMPOWER‐GR
Source: J Cachexia Sarcopenia Muscle. 2023 Dec 20;15(1):352–60. doi: 10.1002/jcsm.13372 (PMC10834324; doi:10.1002/jcsm.13372)
Supplement: Supplementary file 7 — Table S7. Overview of Katz and Lawton and Brody scales at admission and pre‐hospital admission. [file JCSM-15-352-s003.docx]

**Table S7.** Overview of Katz and Lawton and Brody scales at admission and pre-hospital admission

| **Characteristics** | **n** | **At admission** | **Premorbid**^a^ |
| --- | --- | --- | --- |
| Dependent in Katz-ADL, n (%) | 157 |  |  |
| Bathing |  | 99 (63.1) | 23 (14.6) |
| Dressing |  | 101 (64.3) | 16 (10.2) |
| Toileting |  | 78 (49.7) | 157 (100) |
| Transferring |  | 73 (46.5) | 1 (0.6) |
| Continence |  | 36 (22.9) | 6 (3.8) |
| Feeding |  | 7 (4.5) | 3 (1.9) |
| Lawton & Brody-IADL, n (%) | 154 |  |  |
| Telephone |  |  |  |
| Operates on own initiative |  | 98 (63.6) | 116 (75.3) |
| Dials a few well-known numbers |  | 24 (15.6) | 26 (16.9) |
| Answers but does not dial |  | 17 (11.0) | 9 (5.8) |
| Does not use at all |  | 15 (9.7) | 3 (1.9) |
| Shopping |  |  |  |
| Does all shopping independently |  | 5 (3.2) | 84 (54.5) |
| Shops independently for small purchases |  | 18 (11.7) | 28 (18.2) |
| Needs to be accompanied for any shopping trip |  | 29 (18.8) | 13 (8.4) |
| Completely unable to shop |  | 102 (66.2) | 29 (18.8) |
| Food preparation |  |  |  |
| Plans, prepares and serves meals independently |  | 8 (5.2) | 108 (70.1) |
| Prepares adequate meals if supplied with ingredients |  | 31 (20.1) | 9 (5.8) |
| Heats and serves prepared meals |  | 45 (29.2) | 26 (16.9) |
| Needs to have meals prepared and served |  | 70 (45.5) | 11 (7.1) |
| Housekeeping |  |  |  |
| Maintains house alone or with occasional assistance |  | 4 (2.6) | 52 (33.8) |
| Performs light daily task (dishwashing, bed making) |  | 12 (7.8) | 40 (26.0) |
| Performs light daily task but no sufficient |  | 17 (11.0) | 27 (17.5) |
| Needs help with all home maintenance tasks |  | 31 (20.1) | 21 (13.6) |
| Does not participate in any housekeeping tasks |  | 90 (58.4) | 14 (9.1) |
| Laundry |  |  |  |
| Does personal laundry completely |  | 5 (3.2) | 76 (49.4) |
| Launders small items |  | 11 (7.1) | 45 (29.2) |
| All laundry must be done by others |  | 138 (89.6) | 33 (21.4) |
| Mode of transportation |  |  |  |
| Travels independently (public transport/ own car) |  | 5 (3.2) | 79 (51.6) |
| Arranges own taxi but does not use public transport |  | 16 (10.4) | 22 (14.4) |
| Uses public transport when assisted or accompanied |  | 20 (13.0) | 17 (11.1) |
| Travel limited to taxi or car with assistance |  | 83 (53.9) | 30 (19.6) |
| Does not travel at all |  | 30 (19.5) | 5 (3.3) |
| Medication |  |  |  |
| Takes medication in correct dosages and time |  | 71 (46.1) | 131 (85.1) |
| Medication needs to be prepared in advance |  | 69 (44.8) | 19 (12.3) |
| Not capable of dispensing own medication |  | 14 (9.1) | 4 (2.6) |
| Finances |  |  |  |
| Manages finances independently |  | 69 (44.8) | 97 (63.0) |
| Manages day to day purchases but needs help |  | 51 (33.1) | 45 (29.2) |
| Incapable of handling money |  | 34 (22.1) | 12 (7.8) |

All data presented as n (%).^a^ Two weeks prior to acute hospitalization.
